# Acsl4-mediated Lipid Homeostasis Orchestrates Synaptic and Cognitive Plasticity

**DOI:** 10.21203/rs.3.rs-7437638/v1

**Published:** 2026-04-28

**Authors:** Dong Kong, Hu-Song Li, Beikl Liu, Qi-Peng Zhang, Yu Jin, Dwight Chambers, Lesley Colgan, Hannah Kang, Joshua Passarelli, Gabriel Mzaouakk, Senmiao Sun, Yingzi Yang, Clary Clish, Andrew Greenberg, Ryohei Yasuda

**Affiliations:** Boston Children’s Hospital/Harvard Medical School; Boston Children’s Hospital/Harvard Medical School; Boston Children’s Hospital/Harvard Medical School; Nanjing University; Harvard School of Dental Medicine; Boston Children’s Hospital/Harvard Medical School; Max Planck Florida Institute for Neuroscience; Boston Children’s Hospital / Tufts; Boston Children’s Hospital / Northeastern U; Boston Children’s Hospital / Harvard Medical School; Harvard School of Dental Medicine; Broad Institute of Harvard and MIT; HNRCA/Tufts; Max Planck Florida Institute for Neuroscience

## Abstract

Intellectual disability (ID) affects 1–3% of the population, yet effective therapies remain elusive. Dysregulated lipid metabolism has been implicated in ID, but the underlying mechanisms and translational potential are poorly understood. Here, we identify the lipid-metabolizing enzyme acyl-coenzyme A (CoA) synthetase long-chain family member 4 (Acsl4) as a key regulator of synaptic plasticity, engram cell activation, and cognition, in a developmental stage-dependent manner. Neuron-specific Acsl4 knockout mice, which recapitulate key features of human ID, exhibit disrupted diacylglycerol-protein kinase c (PKC) signaling – a pathway essential for memory formation. Lipidomic and transcriptomic profiling reveals a reduction in diacylglycerol species and downregulation of synapse-related genes. Remarkably, restoring expression of the brain-specific Acsl4_B isoform via AAV-mediated gene therapy during development, or pharmacologically activating PKC with Bryostatin 1 – a clinically tested compound – fully rescues cognitive and synaptic deficits. These findings define the pathogenic mechanism of ACSL4-related ID and uncover a therapeutically actionable lipid signaling pathway, providing a framework for targeted intervention in neurodevelopmental disorders involving lipid dysregulation.

Intellectual disability (ID) affects 1–3% of the global population, represents the most frequent disabilities in children, and imposes substantial societal and economic burden^1–5^. Despite its prevalence, most cases of ID are not preventable, and therapeutic options remain limited once the condition is diagnosed^6,7^. Understanding the genetic and molecular mechanisms underlying ID is essential for developing targeted therapies.

Among the diverse causes of ID, dysregulated lipid metabolism has emerged as a critical yet understudied factor^8–12^. The brain, composed of over 60% lipids by dry weight, undergoes dynamic lipid compositional changes, particularly during early development^13–15^. Lipids not only constitute the structural framework of neuron membranes but also serve as energy sources and signaling molecules^16–18^. For example, arachidonic acid (AA), like docosahexaenoic acid (DHA) and eicosapentaenoic acid (EPA)^19^, is biologically active and involved in early brain development, maintains neuronal membrane fluidity in the hippocampus^20^, protects the brain from oxidative stress^21^, and signals many neuronal activities, including calcium dynamics, kinase activities, neurotransmitter uptake, and long-term potentiation (LTP)^22^; excess accumulation of AA is neurotoxic^23, 24^. Therefore, lipids are dynamically regulated in the brain and their dysregulations have been implicated in many neurodevelopmental and neurological disorders, including ID, autism spectrum disorder (ASD), Alzheimer’s disease, and epilepsy^18, 25–27^. Furthermore, ID patients frequently exhibit systemic metabolic abnormalities, including altered serum lipid profiles and body weight irregularities^28–31^, while metabolic disorders like obesity and dyslipidemia are associated with an increased risk of cognitive impairments^32, 33^. Despite these associations, the lipid species and pathways contributing to ID remain poorly understood, largely due to the extreme complexity of brain lipid metabolism.

Long-chain acyl-coenzyme A (CoA) synthetases (ACSLs) are essential in lipid metabolism, adding CoA groups to fatty acids (FAs) to form fatty acyl-CoA, effectively “activating and trapping” FAs within cells^34, 35^. Once activated, acyl-CoAs incorporate into phospholipids, triacylglycerols, cholesterol esters, -oxidation, FA elongation, protein acylation, and can also be cellular signaling molecules^35^. The physiological and pathological roles of ACSLs in the brain, however, are poorly understood^36–40^. Among the five ACSL isozymes in mammals and human, ACSL4 is of particular interest by its substrate preference for polyunsaturated fatty acids (PUFAs), including both AA and EPA^41^. In addition to the ubiquitous variant (ACSL4_U), a second brain-specific isoform for ACSL4 (ACSL4_B) exists from alternative splicing, yet neither their roles in the brain were studied^41–43^. Importantly, null mutations in *ACSL4* have been identified as a leading genetic cause of X chromosome-linked non-syndromic ID in human patients^42, 44, 45^. Unlike syndromic ID, which involves additional physical and neurological abnormalities^46^, non-syndromic ID is characterized by isolated cognitive impairments, thus making ACSL4 an ideal candidate for interrogating the specific impact of lipid dysregulation on neurodevelopmental conditions.

In this study, we performed a comprehensive investigation into Acsl4’s role in neurodevelopment and ID by integrating genetic, molecular, lipidomic, and optic imaging approaches in mice. We demonstrate that Acsl4_B is highly enriched in the hippocampus and dendritic spines, and plays an essential role in synaptic development, function, engram cell activation, and cognition. Neuron-specific *Acsl4* knockout mice exhibit profound cognitive and synaptic deficits, which are fully rescued by re-expressing the Acsl4_B isoform. Our further mechanistic analysis reveals that Acsl4 deficiency disrupts diacylglycerol-PKC signaling, a pathway essential for synaptic plasticity, due to specific reductions in diacylglycerol species. Remarkably, we observe that administering Bryostatin 1, a clinically validated PKC activator, fully reverses the cognitive and synaptic deficits in the *Acsl4* mutated mice and a critical developmental window is essential. Together, these findings establish a mechanistic link between lipid signaling and ID and uncover a viable therapeutic approach for treating ID by targeting lipid-mediated signaling in the developing brain.

## Results

### Characterization of *Acsl4* expression and its isoforms in the mouse brain

**Figure 1:**
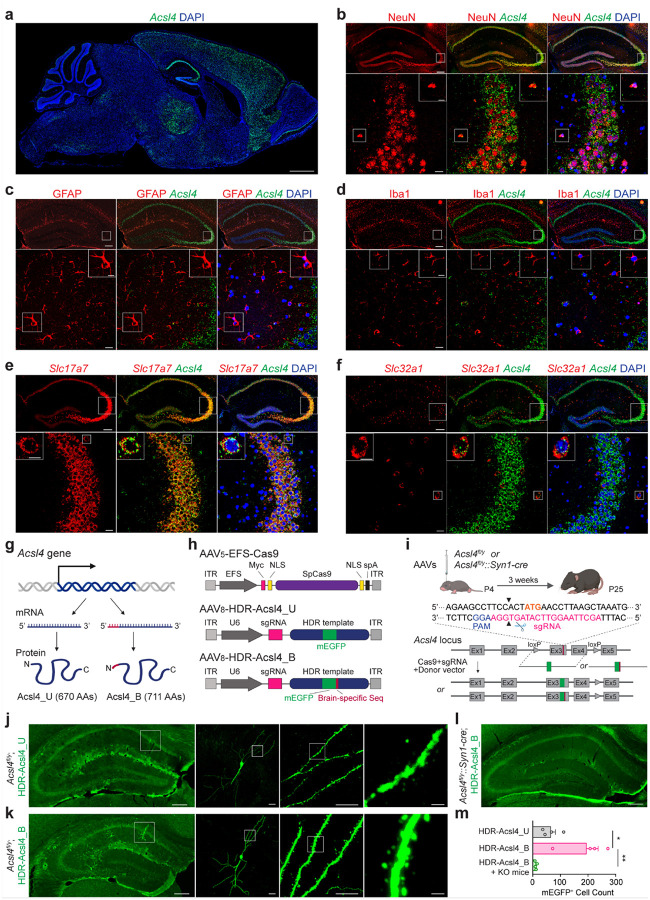
Neuron-specific expression of *Acsl4* and its isoforms in the mouse brain. **a**, RNAscope *in situ* hybridization showing *Acsl4* mRNA expression in a sagittal brain section of wild-type C57BL/6J mouse (male, 8 weeks of age). *Acsl4* mRNA signal was stained in green and nuclei were stained with DAPI. Scale bar: 1 mm. **b-d**, Combination of RNAscope *in situ* hybridization (green) and immunofluorescence (red) to characterize the colocalization of *Acsl4* mRNA with various cell type markers in the hippocampus: anti-NeuN for neurons (**b**); anti-GFAP for astrocytes (**c**); and anti-Iba1 for microglial cells (**d**). Nuclei were stained with DAPI. Scale bars: upper panels, 200 μm; lower panels, 20 μm; insets, 10 μm. **e**, **f**, Dual-color RNAscope *in situ* hybridization to characterize the colocalization of *Acsl4* mRNA (green) with an excitatory neuronal marker *Slc17a7* mRNA (red) (**e**) and an inhibitory neuronal marker *Slc32a1* mRNA (red) (**f**). Nuclei were stained with DAPI. Scale bars: upper panels, 200 μm; lower panels, 20 μm; insets, 10 μm. **g**, A schematic showing alternative splicing of *Acsl4* and its two isoforms in the mouse brain: the ubiquitous isoform, Acsl4_U, and the brain-specific isoform, Acsl4_B. The additional 41 N-terminal amino acids of Acsl4_B are indicated in red. **h**, **i**, Diagrams showing the AAV viral vectors (**h**) and the experimental design of vSLENDR to *in vivo* label Acsl4 isoforms (**i**). EFS, elongation factor-1 short promoter; Myc, c-Myc tag; NLS, nuclear localization signal; spA, synthetic polyadenylation signal; and U6, human U6 polymerase III promoter (**h**). The sgRNA targeting sequence is highlighted in magenta, the sgRNA protospacer-adjacent motif (PAM) sequence in blue, the start codon of *Acsl4* in orange, and the Cas9 cleavage sites are indicated with black arrowheads; green and red blocks denote the sequences of mEGFP and the N-terminal region of Acsl4_B, respectively (**i**). **j-l**, Representative confocal immunostaining images of mEGFP in the hippocampus of *Acsl4*^*fl/y*^ (**j**, **k**) and *Acsl4*^*fl/y*^*::Syn1-cre* (**l**) mice, following vSLENDR-mediated insertion of mEGFP into the locus of *Acsl4* to label Acsl4_U (**j**) and Acsl4_B (**k**, **l**). Scale bar: from left to right, 200 μm, 20 μm, 10 μm, 1 μm (**j**, **k**); and 200 μm (**l**). **m**, Quantification of mEGFP-positive cells. n = 4 mice per group. Cartoons were made using BioRender. *P < 0.05, **P < 0.01. One-way ANOVA test. Data are presented as mean ± SEM. See Source Data for detailed statistics.

*Acsl4* is widely expressed in various tissues and cell types, including the brain, but its regional and cellular expression in the brain has not been fully characterized^41, 47^. To investigate the expression of *Acsl4* in the mouse brain, we performed RNAscope *in situ* hybridization (ISH) using a probe targeting the exons 7–15 of the *Acsl4* gene. We observed that *Acsl4* mRNA is highly expressed in multiple brain regions, with prominent enrichment in the hippocampus, cortex, and thalamus (Fig. 1a). Since the hippocampus plays a central role in learning and memory^48, 49^, we further assessed *Acsl4* expression at the cellular level by combining ISH (to detect *Acsl4* mRNA) with immunofluorescence staining (to label cell-type markers). *Acsl4* mRNA is exclusively expressed in neurons but absent in astrocytes and microglial cells (Fig. 1b–d). Moreover, both excitatory and inhibitory neurons in the hippocampus express *Acsl4*, as demonstrated by co-labeling with *Slc17a7* and *Slc32a1* mRNA (Fig. 1e, f).

To assess the expression of Acsl4 isoforms in the mouse brain *in vivo*, the ubiquitous Acsl4_U and the brain-specific Acsl4_B (Fig. 1g), we employed a method based on virus-mediated single-cell labeling of endogenous proteins via homology-directed repair (vSLENDR)^50^, which enables precise tagging of endogenous proteins through CRISPR/Cas9-mediated homology-directed repair (HDR) by delivering three components into the same cells: a single-guide RNA (sgRNA), a donor template DNA, and *Streptococcus pyogenes* Cas9 (spCas9) (Fig. 1h). Specifically, we used a dual adeno-associated virus (AAV) system for vSLENDR, in which one AAV expressed spCas9 under the control of constitutive elongation factor 1α (EFS) promoter (AAV_5_-EFS-Cas9), while the second AAV contained an sgRNA targeting the start codon of *Acsl4* and an HDR template encoding mEGFP sequences flanked by ~1 kb of *Acsl4* homology arms (AAV_8_-HDR-Acsl4_U or AAV_8_-HDR-Acsl4_B) (Fig. 1h). This system was injected into the hippocampus of *Acsl4*-floxed (*Acsl4*^*fl/y*^) or neuronal *Acsl4* knockout (*Acsl4*^*fl/y*^*::Syn1-cre*) male mice (Fig. 1i) generated by crossing female *Acsl4*^*fl/fl*^ mice with male *Syn1-cre* transgenic mice, in which Cre recombinase is selectively expressed in neurons^51^. Since HDR is more efficient in dividing cells^52^, we performed postnatal day 4 (P4) injections to maximize genome integration efficiency. Three weeks post-injection, strong mEGFP signals were detected in hippocampal neurons of *Acsl4*^*fl/y*^ mice injected with AAV_5_-EFS-Cas9 and AAV_8_-HDR-Acsl4_U or AAV_8_-HDR-Acsl4_B viruses, confirming successful genome integration. While both Acsl4_U and Acsl4_B localized to neuronal cell bodies, Acsl4_B was distinctly enriched in dendrites and spines (Fig. 1j, k). In contrast, only background-level fluorescence was detected in *Acsl4*^*fl/y*^*::Syn1-cre* mice, where Cre-mediated deletion of exons 3 and 4 disrupted HDR, preventing mEGFP tagging (Fig. 1l). Notably, the number of mEGFP-positive neurons was significantly higher in *Acsl4*^*fl/y*^ mice injected with AAV_8_-HDR-Acsl4_B compared to AAV_8_-HDR-Acsl4_U (Fig. 1m), consistent with prior reports indicating higher *Acsl4_B* transcripts in the brain^43^. The near absence of mEGFP-positive neurons in *Acsl4*^*fl/y*^*::Syn1-cre* mice confirmed that the observed fluorescence originated specifically from mEGFP-tagged Acsl4_U or Acsl4_B rather than non-specific integration. Collectively, these findings establish that Acsl4 is neuron-specific in the brain, with Acsl4_B predominantly localized to dendritic compartments, indicating a potential role in synaptic plasticity and neurotransmission.

### Neuronal *Acsl4* deficiency and cognitive deficits

**Figure 2:**
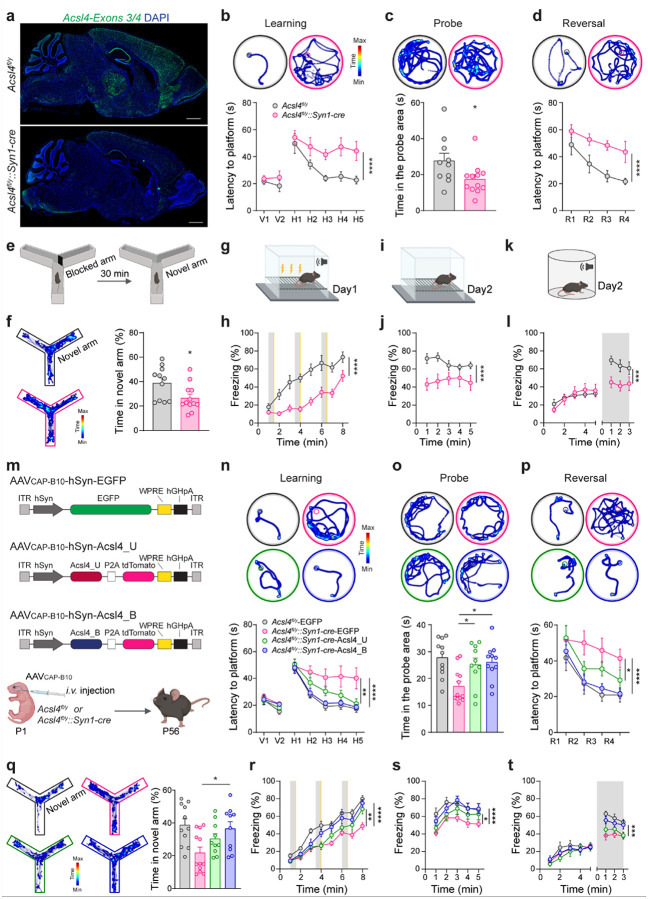
Cognitive deficits by neuron-specific deletion of *Acsl4*. **a**, RNAscope *in situ* hybridization showing *Acsl4* mRNA expression in the brain slices of *Acsl4*^*fl/y*^ and *Acsl4*^*fl/y*^*::Syn1-cre* mice. Nuclei were stained with DAPI. Scale bar: 1 mm. **b-d**, Morris water maze tests. Representative traces (H5) and time spent before reaching the visible (V1 and V2) or hidden (H1, H2, H3, H4, and H5) platform during the training phase (**b**). Representative traces and time spent in the correct quadrant during the probe test phase (**c**). Representative traces (R4) and time spent before reaching the hidden (R1, R2, R3, and R4) platform during the reversal phase (**d**). n = 10 to 12 mice per group. **e**, **f**, Y-maze tests. Experimental scheme (**e**). Representative traces and the percentage of time spent in the novel arm (**f**). n = 11 to 12 mice per group. **g-l**, Fear conditioning tests. Schematics of fear conditioning during training (**g**), same-context test (**i**), and auditory test phases (**k**). Freezing levels during training sessions (**h**), same-context test (**j**), and auditory test in a novel context (**l**). Gray bars indicate tone presentation, and yellow lines indicate shock delivery. n = 10 mice per group. **m**, Schematic of AAV_CAP-B10_ vectors and experimental diagram. **n-p**, Morris water maze tests following re-expression of Acsl4 isoforms. Representative traces (H5) and time spent before reaching the visible (V1 and V2) or hidden (H1, H2, H3, H4, and H5) platform during the training phase (**n**). Representative traces and time spent in the correct quadrant during the probe test phase (**o**). Representative traces (R4) and time spent before reaching the hidden (R1, R2, R3, and R4) platform during the reversal phase (**p**). n = 10 to 12 mice per group. **q**, Y-maze tests following re-expression of Acsl4 isoforms and representative traces and the percentage of time spent in the novel arm. n = 10 to 12 mice per group. **r-t**, Fear conditioning tests following re-expression of Acsl4 isoforms. Freezing levels during the training sessions (**r**), same-context test (**s**), and auditory test in a novel context (**t**). n = 10 to 11 mice per group. Cartoons were made using BioRender. *P < 0.05, **P < 0.01, ***P < 0.001, ****P < 0.0001. Two-way ANOVA test (**b**, **d**, **h**, **j**, **l**, **n**, **p**, **r-t**), one-way ANOVA test (**o**, **q**) or two-tailed unpaired t-test (**c**, **f**). Data are presented as mean ± SEM. See Source Data for detailed statistics.

To investigate whether *Acsl4* is required for maintaining cognitive functions, we examined *Acsl4*^*fl/y*^*::Syn1-cre* male mice, a neuron-specific *Acsl4* knockout mouse model, and compared them to their littermate controls (*Acsl4*^*fl/y*^). To confirm *Acsl4* deletion, RNAscope ISH targeting exons 3 and 4 of *Acsl4* revealed a near-complete loss of *Acsl4* mRNA in the brain of *Acsl4*^*fl/y*^*::Syn1-cre* mice (Fig. 2a). Despite this, these mice appeared grossly normal, displaying normal brain weight (Extended Data Fig. 1a, b), intact brain structure (Extended Data Fig. 1c), and unaltered neuronal, astrocytic, and microglial morphology (Extended Data Fig. 1d-f). Their body length and weight growth were also comparable to controls (Extended Data Fig. 1g, h).

We next performed behavioral assays to evaluate cognitive function. In the Morris water maze (MWM) tests, *Acsl4*^*fl/y*^*::Syn1-cre* mice exhibited significantly longer latencies to locate the hidden platform during both the learning and reversal phases (Fig. 2b, d). They also spent less time in the correct quadrant during the probe phase compared to *Acsl4*^*fl/y*^ control mice (Fig. 2c), indicating long-term spatial learning and memory deficits. Similarly, in the Y-maze tests, knockout mice spent less time exploring the novel arm than controls (Fig. 2e, f), suggesting impaired short-term spatial memory. The fear conditioning tests further confirmed cognitive impairments, as *Acsl4*^*fl/y*^*::Syn1-cre* mice showed significantly reduced freezing responses during both conditioning (Fig. 2g, h) and contextual and cued recall phases (Fig. 2i–l), reflecting deficits in associative fear learning and memory.

To exclude potential confounding factors, we assessed sensory and motor functions. Hot plate and startle response tests showed no significant differences in pain sensitivity or auditory response between knockout and control mice (Extended Data Fig. 1i–l). Prepulse inhibition (PPI) tests also revealed normal sensorimotor gating (Extended Data Fig. 1m), confirming that the observed cognitive impairments were not due to sensory deficits.

As *Acsl4* is X-linked^41^, we also characterized female neuronal *Acsl4* knockout (*Acsl4*^*fl/fl*^*::Syn1-cre*) mice. Like males, female knockouts exhibited normal brain weight, structure, and cellular morphology (Extended Data Fig. 2a-f) and no changes in body weight (Extended Data Fig. 2g). However, they displayed the same cognitive impairments in MWM (Extended Data Fig. 2h–j), Y-maze (Extended Data Fig. 2k), and fear conditioning tests (Extended Data Fig. 2l-n). Their sensory functions remained intact (Extended Data Fig. 2o-q), confirming that the role of *Acsl4* in cognition is not sex-specific.

### Restoration of Acsl4 isoforms in the knockout mice

Since both Acsl4_U and Acsl4_B are expressed in the brain and deletion of exons 3 and 4 disrupts both variants^42, 43^, which isoform (or both) is required for cognitive function is undetermined; their distinct subcellular localizations may also suggest functional differences (Fig. 1j, k). To assess this, we further conducted isoform-specific rescue experiments. Using AAV vectors driven by the human synapsin-1 (hSyn) promoter, we separately expressed Acsl4_U or Acsl4_B in *Acsl4*^*fl/y*^*::Syn1-cre* mice. These vectors carried tdTomato-tagged Acsl4 isoforms linked by a self-cleaving P2A peptide (Fig. 2m). To ensure efficient brain-wide expression, we packaged the vectors into a recently developed AAV capsid variant, AAV_CAP-B10_^53^, with superior blood-brain barrier penetration and reduced liver transduction compared with other AAV serotypes like AAV_9_ (Extended Data Fig. 3a-c). P1 intravenous injections of these viruses resulted in widespread neuronal expression in the adult mice while minimizing peripheral expression (Extended Data Fig. 3d, e). Behavioral analyses in adulthood showed that Acsl4_B fully rescued cognitive deficits, restoring performance in the MWM, Y-maze, and fear conditioning tests (Fig. 2n–t). In contrast, Acsl4_U provided only partial rescue in *Acsl4*^*fl/y*^*::Syn1-cre* mice and exhibited significantly more residual impairments than Acsl4_B group, underscoring that Acsl4_B is the functionally essential isoform for cognitive function.

### Necessity of Acsl4 in the critical developmental period

**Figure 3:**
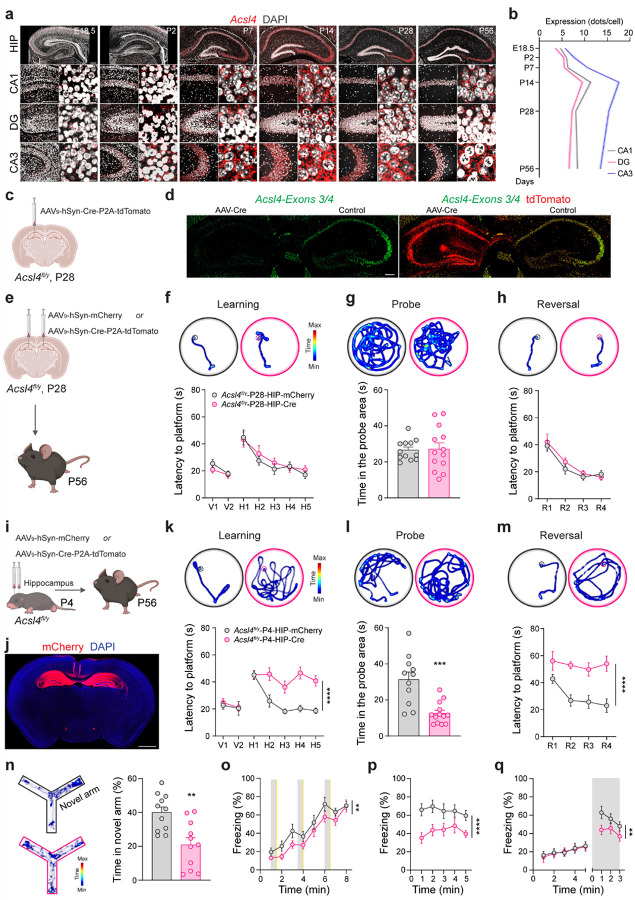
Early postnatal expression of *Acsl4* in neurodevelopment and cognition. **a**, **b,** Representative images (**a**) and quantification of *Acsl4* mRNA expression in the hippocampus during developmental stages (**b**). n = 4 slices from 4 mice. Scale bar: 200 μm (HIP), 100 μm (CA1, DG, and CA3), 20 μm (insets of CA1, DG, and CA3). **c**, **d**, Schematic of unilateral stereotactic AAV injection in P28 *Acsl4*^*fl/y*^ mice (**c**) and ISH of *Acsl4* mRNA expression confirming Cre-mediated deletion of the gene (**d**). Scale bar: 200 μm. **e-h**, Experimental diagram and Morris water maze tests following AAV-Cre mediated deletion of *Acsl4* in the hippocampus in P28 *Acsl4*^*fl/y*^ mice. n = 12 to 13 mice per group. **i-q**, Experimental diagram (**i**), immunofluorescence against mCherry (**j**), Morris water maze tests (**k-m**), Y-maze tests (**n**), and fear conditioning tests following AAV-Cre mediated deletion of *Acsl4* in the hippocampus in P4 *Acsl4*^*fl/y*^ mice (**o-q**). n = 9 to 13 mice per group. Cartoons were made using BioRender. **P < 0.01, ***P < 0.001, ****P < 0.0001. Two-way ANOVA test (**f**, **h**, **k**, **m**, **o-q**) or two-tailed unpaired t-test (**g**, **l**, **n**). Data are presented as mean ± SEM. See Source Data for detailed statistics.

Neurodevelopment is marked by a period of heightened plasticity, during which key processes such as synaptogenesis, neuronal maturation, axon elongation, and neural network formation shape long-term cognitive function^54–56^. Disruptions during this critical time window can lead to lasting deficits, underscoring its importance in establishing normal brain functions^57^. To determine whether *Acsl4* plays a role in this critical developmental period, we first examined *Acsl4* expression across brain regions during development. *Acsl4* expression surged from birth to postnatal day 14 (P14) in the cortex, thalamus, and particularly in the hippocampus, followed by a gradual decline into adulthood (Extended Data Fig. 4a and Fig. 3a, b).

To explore the spatiotemporal role of Acsl4 in cognitive development, we used an AAV_9_-hSyn-Cre-P2A-tdTomato vector to delete *Acsl4* in the hippocampus at specific developmental stages, via stereotaxic injection of the virus into *Acsl4*^*fl/y*^ male mice. ISH confirmed significant *Acsl4* mRNA reduction in the unilateral hippocampus injected with the virus, compared to the control non-injected side (Fig. 3c, d). We performed bilateral hippocampal injections of AAV_9_-hSyn-Cre-P2A-tdTomato virus at P28 (*Acsl4*^*fl/y*^-P28-HIP-Cre), using AAV_9_-hSyn-mCherry virus as a control (*Acsl4*^*fl/y*^-P28-HIP-mCherry) (Fig. 3e). Four weeks later, MWM tests revealed no significant differences in spatial learning and memory between *Acsl4*^*fl/y*^-P28-HIP-Cre and *Acsl4*^*fl/y*^-P28-HIP-mCherry mice (Fig. 3f–h), indicating that *Acsl4* expression after P28 is dispensable for cognitive function.

Then, we performed a similar injection of the viruses into the hippocampus of P4 *Acsl4*^*fl/y*^ mice to delete the gene at an early stage (Fig. 3i, j). Behavioral assessments in adulthood revealed that *Acsl4*^*fl/y*^-P4-HIP-Cre mice exhibited significant impairments in MWM and Y-maze tests (Fig. 3k–n), closely resembling the cognitive deficits observed in *Acsl4*^*fl/y*^*::Syn1-cre* knockout mice. In the fear conditioning tests, *Acsl4*^*fl/y*^-P4-HIP-Cre mice exhibited mildly impaired fear acquisition (Fig. 3o) but severely disrupted fear memory (Fig. 3p, q), suggesting that while *Acsl4* in other brain regions contribute to fear acquisition, hippocampal *Acsl4* is crucial for fear memory regulation.

To determine whether Acsl4 overexpression enhances cognitive function, we bilaterally injected AAV_9_-hSyn-Acsl4_B virus into the hippocampus of P4 wild-type (WT) *C57BL/6J* mice (Extended Data Fig. 4b). However, behavioral testing in adulthood revealed no significant cognitive improvements compared to control mice injected with AAV_9_-hSyn-mCherry virus (Extended Data Fig. 4c-i), suggesting that overexpressing Acsl4_B is insufficient to enhance normal learning and memory. Collectively, these findings establish a temporal and spatial requirement for Acsl4 in cognitive development, with its early hippocampal expression being critical for learning and memory.

### Acsl4 deficiency and the hippocampal transcriptome

**Figure 4:**
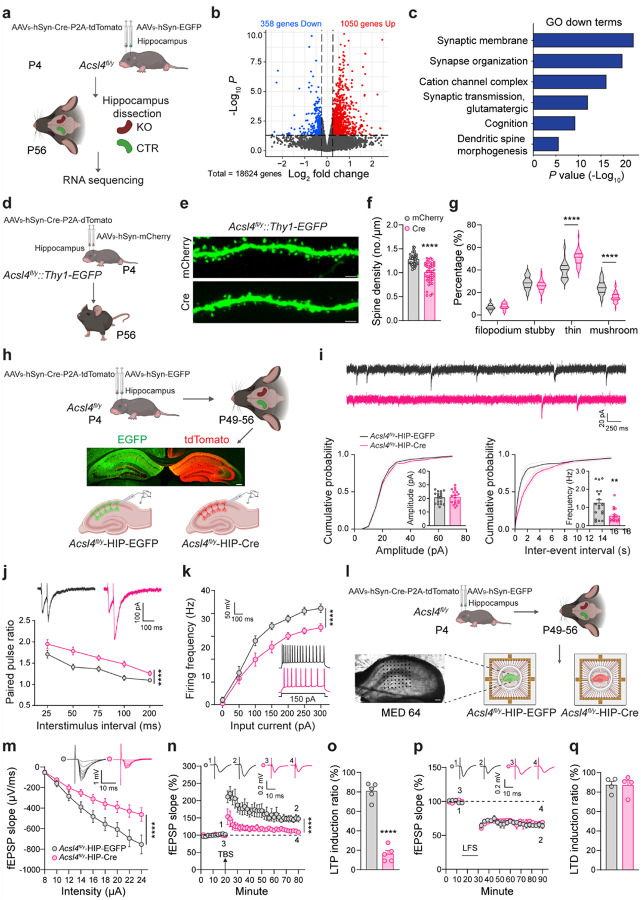
Loss of *Acsl4* impairs dendritic spine maturation, neuronal intrinsic excitability, and synaptic plasticity in the hippocampus. **a**, Flowchart depicting the bulk RNA-seq workflow using hippocampal tissues from *Acsl4* knockout (KO) and control (CTR) hemispheres. One hippocampus was injected with AAV-Cre to delete *Acsl4* (KO), while the contralateral hippocampus received AAV-EGFP injection as the control (CTR). **b**, Volcano plot showing differentially expressed genes in RNA-seq analysis of the hippocampus between CTR and KO (KO vs. CTR). Differentially expressed genes are defined as those with *P* < 0.05 and log_2_(fold change) > 0.25 or < −0.25. Red indicates upregulated genes, while blue indicates downregulated genes. **c**, Gene ontology (GO) analysis highlighting terms significantly enriched in the downregulated genes. GO terms with *P* < 0.05 were considered significant. **d**, Schematic illustration of the experimental design for neuronal imaging. **e-g**, Effects of *Acsl4* deletion on CA1 neuron dendritic spines. Representative images of dendritic spines (**e**). Scale bar: 2 μm. Quantification of spine density (**f**) and percentage of spine types (**g**). n = 30 to 44 cells from 3 mice per group. **h**, Schematic of the experimental design for patch-clamp recordings. Scale bar: 200 μm. **i**, Representative traces and cumulative distribution plots of amplitudes and frequency of mEPSCs. Inset bar graphs show average for each. n = 18 cells from 4 mice per group. **j,** Representative traces and quantification of paired-pulse ratio recordings. n = 16 cells from 4 mice per group. **k**, Representative traces and intensity-dependent increase in firing frequency. n = 15 cells from 4 mice per group. **l**, Schematic diagram of the experimental design for MED64 recording and a brightfield image showing the placement of the MED64 probe relative to the hippocampus slice. Scale bar: 200 μm. **m**, Representative traces and input-output curves of the fEPSP slope in response to a series of graded stimulation intensities. n = 6 slices from 3 mice per group. **n**, **o**, TBS-induced LTP at Schaffer collateral (SC) to CA1 synapses. Representative fEPSP traces at the time points indicated and a summary plot of fEPSP slopes (**n**). The arrow indicates TBS application. Induction ratios of CA1 LTP (**o**). n = 5 slices from 5 mice per group. **p**, **q**, LFS-induced LTD at Schaffer collateral (SC) to CA1 synapses. Representative fEPSP traces at the time points indicated and a summary plot of fEPSP slopes (**p**). The bar indicates LFS application. Induction ratios of CA1 LTD (**q**). n = 4 slices from 4 mice per group. Cartoons were made using BioRender. **P < 0.01, ****P < 0.0001. Two-tailed unpaired t-test (**f**, **i**-amplitude, **o**, **q**), two-tailed Mann Whitney test (**i**-frequency), or two-way ANOVA test (**g**, **j**, **k**, **m**, **n**, **p**). Data are presented as mean ± SEM. See Source Data for detailed statistics.

To explore the molecular and neurobiological mechanisms underlying *Acsl4*-mediated cognitive regulation, we conducted transcriptomic profiling of the hippocampus following *Acsl4* deletion. Specifically, we injected AAV_9_-hSyn-Cre-P2A-tdTomato virus into the hippocampus of P4 *Acsl4*^*fl/y*^ mice to delete *Acsl4* (KO) and collected hippocampal tissues at P56 for bulk RNA sequencing (RNA-seq). The contralateral hippocampus, injected with AAV_9_-hSyn-EGFP virus, served as the control (CTR) within the same mouse, minimizing inter-individual variability (Fig. 4a).

Differential gene expression analysis revealed 1,408 genes significantly altered in Acsl4-deficient hippocampi, with 358 genes downregulated and 1,050 genes upregulated (*P* < 0.05, log_2_FC > 0.25 or < −0.25) (Fig. 4b). Gene Ontology (GO) enrichment analysis showed that the downregulated genes were mostly associated with synaptic function and plasticity, including those involved in synaptic membrane integrity (such as *Syt7* and *Gria3*), pre- and post-synaptic organization (such as *Shank3* and *Shank1*), cation channel complex (such as *Trpc5* and *Scn1a*), glutamatergic neurotransmission, dendritic spine morphogenesis (such as *Grin1*, *Grm8*, *Ephb2*, and *Epha5*), and those known to affect cognition via modulating synaptic plasticity (such as *Syngap1* and *Prrt1*), indicating impaired synaptic integrity and function following *Acsl4* deletion (Fig. 4c and Extended Data Fig. 5a-f).

Interestingly, many of the upregulated genes were linked to immune and inflammatory responses, including genes involved in microglial activation (such as *Cx3cr1* and *Itgam*), cytokine signaling (such as *Tlr2/3/4/7*, *Il18*, *Il33*, and *Il6ra*), and cell activation (such as *Ptprc*, *Slc11a1*, and *CD84*) (Extended Data Fig. 6a-f). This suggests that *Acsl4* deletion not only disrupts synaptic structure but also triggers inflammatory responses, which may further contribute to neuronal dysfunction. Several of the upregulated genes, such as *Trem2*, *Il33*, and *C3*, are key components of the phagocytic signaling pathway, which has been implicated in synapse pruning and neuroinflammation in neurodevelopmental disorders^58–60^. In addition, many genes involved in various aspects of lipid metabolism were also upregulated (Extended Data Fig. 6g-m). Since lipids are essential for synaptic membrane organization and function, these changes may reflect a compensatory response to Acsl4 loss or a direct disruption of lipid-regulated neuronal processes.

### Acsl4 and the hippocampal synaptic plasticity

Since RNA-seq analysis suggested that Acsl4 regulates synaptic function, we next examined its role in synapse morphology and electrophysiological properties. To assess dendritic spine morphology, we crossed *Thy1-EGFP* mice^61^ with *Acsl4*^*fl/y*^ mice to obtain *Acsl4*^*fl/y*^*::Thy1-EGFP* mice and to achieve sparse Golgi-like fluorescent labeling of hippocampal neurons. We then stereotaxically injected AAV_9_-hSyn-Cre-P2A-tdTomato into the hippocampus of P4 *Acsl4*^*fl/y*^*::Thy1-EGFP* mice to delete *Acsl4* and used similarly injected AAV_9_-hSyn-mCherry in the contralateral hippocampus as the control, followed by immunofluorescent staining against EGFP and mCherry/tdTomato (Fig. 4d and Extended Data Fig. 7a, b). At adulthood, Cre/tdTomato-positive CA1 pyramidal neurons exhibited no significant changes in total dendritic length, dendritic branching, or Sholl analysis compared to mCherry-positive control neurons (Extended Data Fig. 7c-f), suggesting that *Acsl4* deletion does not affect dendritic architecture. However, dendritic spine density was significantly reduced in Cre-positive neurons (Fig. 4e, f). Moreover, the proportion of mature mushroom spines decreased, whereas the proportion of thin spines increased (Fig. 4g), indicating a shift toward immature spine morphology following *Acsl4* deletion.

Given these structural impairments, we next assessed synaptic functions of these neurons using whole-cell patch-clamp recordings from hippocampal slices of *Acsl4*^*fl/y*^ mice, following unilateral injection of AAV_9_-hSyn-Cre-P2A-tdTomato (*Acsl4*^*fl/y*^-HIP-Cre), with the contralaterally injected AAV_9_-hSyn-EGFP within the same mouse as the control (Fig. 4h). We observed a significant reduction in miniature excitatory postsynaptic currents (mEPSC) frequency but not the amplitude (Fig. 4i), suggesting that *Acsl4* deletion weakens glutamatergic neurotransmission without affecting postsynaptic receptor function. This was further supported by an increased paired-pulse ratio (PPR) in *Acsl4*-deficient neurons, indicating impaired presynaptic neurotransmitter release probability (Fig. 4j). Additionally, current-clamp recordings revealed reduced frequency of action potential firing in response to current injection in *Acsl4*-deleted neurons (Fig. 4k), demonstrating the necessity of Acsl4 for intrinsic neuronal excitability. These hippocampal synaptic and neuronal impairments following *Acsl4* deletion were also validated in *Acsl4*^*fl/y*^*::Syn1-cre* knockout mice (Extended Data Fig. 8a-l). Of interest, no changes were observed in either the frequency or amplitude of miniature inhibitory postsynaptic currents (mIPSC) (Extended Data Fig. 8j), suggesting a unique role of Acsl4 in glutamatergic synapses, but not in GABAergic ones.

To investigate the impact of Acsl4 deletion on long-term synaptic plasticity, we performed multielectrode array (MED64)^62, 63^ recordings in the hippocampal slices (Fig. 4l). Basal synaptic transmission, assessed by Schaffer collateral stimulation and field excitatory postsynaptic potentials (fEPSP) in CA1 neurons, was significantly reduced in Acsl4-deleted neurons (Fig. 4m), consistent with the patch-clamp data. Furthermore, theta burst stimulation (TBS)-induced long-term potentiation (LTP) was dramatically impaired: in control slices (*Acsl4*^*fl/y*^-HIP- EGFP), LTP was successfully induced in 70 activated channels, whereas in *Acsl4*^*fl/y*^-HIP-Cre slices, LTP induction occurred in only 15 channels (Extended Data Fig. 9a-e). Quantification of LTP magnitude showed a significantly blunted potential curve and reduced LTP induction ratio in *Acsl4*-deficient slices (Fig. 4n, o). By contrast, long-term depression (LTD), induced by low frequency stimulation (LFS), remained unaffected (Fig. 4p, q and Extended Data Fig. 9f-i), indicating that Acsl4 selectively regulates LTP but not LTD. These findings were also similarly validated in *Acsl4*^*fl/y*^*::Syn1-cre* knockout mice (Extended Data Fig. 10a-n).

### Acsl4 in the hippocampal engram cells

**Figure 5:**
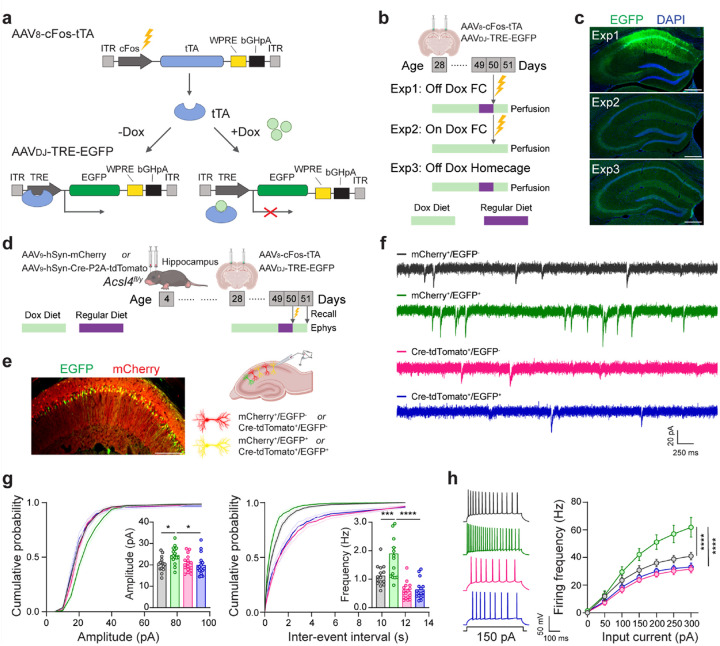
*Acsl4* is required for hippocampal engram cell activation. **a**, Schematic illustration of dual AAVs composed of Tet-Off system for labeling engram cells. **b**, **c**, Experimental design (**b**) and representative immunofluorescent images to validate the Tet-Off system for labeling fear memory engram cells in the hippocampus (**c**). FC, fear conditioning. Nuclei were stained with DAPI. Scale bar: 200 μm. **d-h**, Experimental flow diagram (**d**), immunostaining showing labeled engram cells (EGFP, green) and AAV-infected hippocampal neurons (mCherry, red) (**e**), representative traces and quantification of mEPSCs (**f**, **g**), and representative traces and quantification of intensity-dependent firing frequency (**h**). Scale bar: 200 μm. n = 12 to 17 cells from 6 mice per group. Cartoons were made using BioRender. *P < 0.05, ***P < 0.001, ****P < 0.0001. One-way ANOVA test (**g**) or two-way ANOVA test (**h**). Data are presented as mean ± SEM. See Source Data for detailed statistics.

Engram cells in the hippocampus and other brain regions provide the neural underpinnings of memory, including its encoding, consolidation, retrieval, and forgetting^64–67^. To investigate whether Acsl4 regulates cognitive functions by directly modulating the activities of hippocampal engram cells, we employed a dual-AAV-Tet-Off system^68–70^ to selectively label engram cells during contextual fear conditioning tests (Fig. 5a). To validate this approach, we injected AAVs encoding EGFP under Tet-Off regulation into the hippocampus of P28 WT mice and compared EGFP expression under three different conditions (Fig. 5b). In mice maintained on a doxycycline (Dox)-free diet for one day prior to fear conditioning at P50 (Off Dox FC), we observed robust EGFP in the CA1 hippocampal region. In contrast, only minimal background EGFP expression was observed in the homecage controls (Off Dox Homecage), confirming that EGFP labeling was specific to activated engram cells. Additionally, no EGFP expression was detected in Dox-treated mice, even after fear conditioning (On Dox FC) (Fig. 5c). These results demonstrate that the Tet-Off system reliably and specifically labels engram cells activated during fear conditioning learning.

To determine whether Acsl4 regulates engram cell activity, we injected AAV_9_-hSyn-Cre-P2A-tdTomato into the hippocampus of P4 *Acsl4*^*fl/y*^ mice to delete *Acsl4* and introduced the Tet-Off system at P28 to label engram cells during fear conditioning performed at P50. AAV_9_-hSyn-mCherry injected P4 *Acsl4*^*fl/y*^ mice served as the control (Fig. 5d). Whole-cell patch-clamp recordings were performed in EGFP^+^/engram and EGFP^−^/non-engram neurons following the fear memory recall tests (Fig. 5e). In control mice, engram cells exhibited increased mEPSC amplitude and frequency compared to nonengram neurons, reflecting enhanced excitatory synaptic transmission. Additionally, engram cells displayed increased action potential firing in response to current injections, indicating elevated intrinsic excitability. However, these enhancements were significantly attenuated in Acsl4-deficient mice injected with AAV_9_-hSyn-Cre-P2A-tdTomato (Fig. 5f–h). These findings demonstrate that Acsl4 is essential for the synaptic potentiation and intrinsic excitability of hippocampal engram cells, which mediate fear memory storage and retrieval.

### Acsl4 deficiency and the hippocampal lipidomic analysis

**Figure 6:**
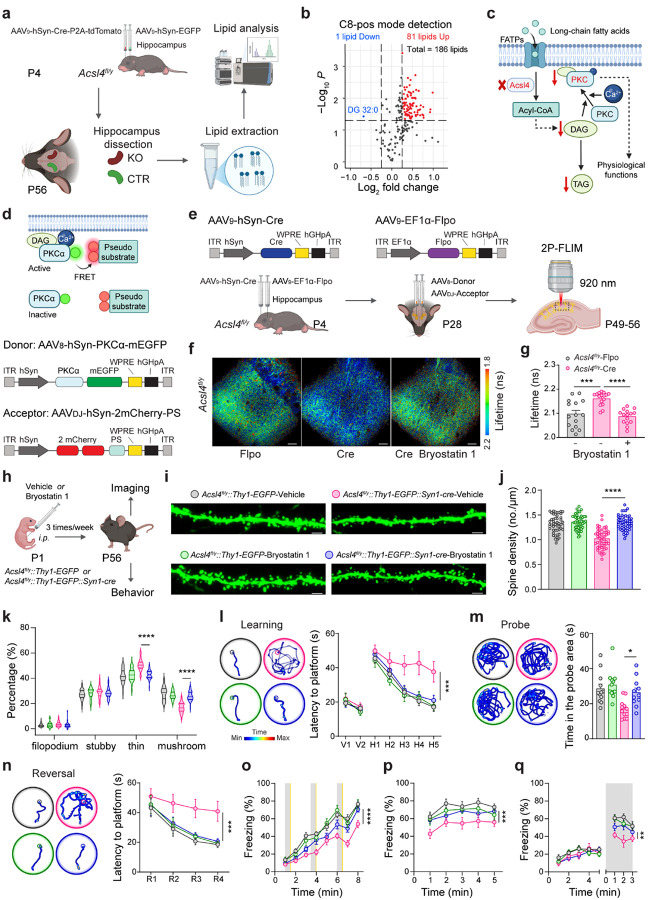
Loss of *Acsl4* impairs DAG-PKC signaling activation. **a**, **b**, Experimental flow diagram (**a**) and volcano plot of lipids in C8-positive ion mode analysis from lipidomic studies on hippocampal tissues from *Acsl4* deletion (KO) and control (CTR) contralateral hemispheres (**b**). Differentially accumulated lipids are defined as those with *P* < 0.05 and log_2_(fold change) > 0.25 or < −0.25. Red indicates upregulated lipids, while blue indicates downregulated lipids. **c**, Hypothetic diagram of Acsl4’s role in PKC signaling activation. FATPs, fatty acid transport proteins. DAG, diacylglycerol. TAG, triacylglycerol. PKC, protein kinase C. **d**, **e**, Schematic of PKCα 2-photon fluorescence lifetime imaging (2P-FLIM) sensor (**d**) and experimental workflow diagram (**e**). **f**, **g**, Effects of Acsl4 deficiency or Bryostatin 1 on PKCα activity. Representative images (**f**) and quantification of fluorescent lifetime from hippocampal CA1 region expressing PKCα sensor in control (*Acsl4*^*fl/y*^-Flpo), *Acsl4* knockout (*Acsl4*^*fl/y*^-Cre), or *Acsl4* knockout mice with Bryostatin 1 treatment (*Acsl4*^*fl/y*^-Cre+Bryostatin 1) (**g**). Scale bar: 20 μm. n = 15 slices from 5 mice per group. **h-k**, Experimental design (**h**), representative images of dendritic spines (**i**), quantification of spine density (**j**), percentage of spine types (**k**). Scale bar: 2 μm. n = 48 cells from 4 mice per group. **l-q**, Morris water maze tests (**l-n**), and fear conditioning tests (**o-q**) from control or Acsl4 neuron-specific knockout mice following vehicle or Bryostatin 1 treatment. n = 11 to 12 mice per group. Cartoons were made using BioRender. * *P* < 0.05, **P < 0.01, ***P < 0.001, ****P < 0.0001. One-way ANOVA test (**g**, **j**, **m**) or two-way ANOVA test (**k**, **l**, **n**, **o-q**). Data are presented as mean ± SEM. See Source Data for detailed statistics.

As Acsl4 is a key enzyme in lipid biosynthesis, we hypothesized that its loss leads to disruptions in specific lipid species involved in neuronal regulation. To profile such lipids and further elucidate the molecular mechanisms by which Acsl4 regulates synapse development and function, we conducted lipidomic analyses of the hippocampus following AAV_9_-hSyn-Cre-P2A-tdTomato virus-mediated *Acsl4* deletion (Fig. 6a). Similar as the RNA-seq study (Fig. 4a), contralateral side of the hippocampus injected with AAV_9_-hSyn-EGFP virus within the same mouse were taken as the control. Lipidomic profiling revealed significant alterations in lipid composition in *Acsl4*-deficient hippocampi compared to controls, with 81 species significantly upregulated and one notably reduced (e.g. diacylglycerol (DAG) 32:0) (Fig. 6b and Extended Data Fig. 11a). Among the upregulated lipid species, arachidonic acid (AA) and eicosapentaenoic acid (EPA), two preferred substrates of Acsl4^47^, were both found to be accumulated in the hippocampus following *Acsl4*-deletion, confirming the knockout efficiency (Extended Data Fig. 11b). DAGs function as critical endogenous co-activators of protein kinase C (PKC), a key signaling molecule involved in disparate physiological processes, including synaptic development and plasticity^71–74^. Among mammalian PKC isoforms, PKCα is a conventional isoform that requires both calcium ions and DAGs for its activation, and has been shown to be essential for hippocampal synaptic plasticity and development^72, 75^. We then hypothesized that loss of Acsl4 leads to reduced DAG 32:0 levels, which in turn attenuates PKCα activity during neurodevelopment, ultimately impairing synaptic function and cognition (Fig. 6c).

To assess hippocampal PKCα activity following *Acsl4* deletion, we employed a PKCα Förster resonance energy transfer (FRET) sensor to enable two-photon fluorescence lifetime imaging (2P-FLIM)^72^ (Fig. 6d). We first validated the PKCα FLIM-FRET sensor in HEK293T cells following plasmid co-transfection, and observed a significant decrease in fluorescence lifetime after PKCα activation by both phorbol 12,13-dibutyrate (PDBu) and ionomycin (Extended Data Fig. 12a-c). Next, we constructed two AAV vectors carrying the donor and acceptor components of the PKCα sensor, respectively (AAV_8_-hSyn-PKCα-EGFP and AAV_DJ_-hSyn-2mCherry-PS), stereotaxically injected them into the hippocampus of P4 WT *C57BL/6J* mice, and tested their functionality in acutely-prepared hippocampal brain slices 6–7 weeks post injection (Extended Data Fig. 12d). In the brain slices transfected with both the donor and acceptor AAVs, we observed significantly decreased fluorescence lifetime in the hippocampal CA1 neurons upon PDBu and ionomycin treatment. However, in the brain slices transfected with the donor AAV only, such a reduction was absent (Extended Data Fig. 12e, f), confirming that the PKCα sensor reliably detects PKCα activity in hippocampal neurons.

To determine whether PKCα activation is impaired in the *Acsl4*-deficient hippocampus, we stereotaxically injected AAV_9_-hSyn-Cre or AAV_9_-EF1α-Flpo virus into the hippocampus of P4 *Acsl4*^*fl/y*^ mice, followed by a secondary injection of the sensor AAVs at P28. 3–4 weeks later, hippocampal brain slices were prepared for 2P-FLIM analysis (Fig. 6e). We observed a notably increased fluorescence lifetime in the *Acsl4*-deficient hippocampal neurons, compared to the control neurons transfected by AAV_9_-hSyn-Flpo (Fig. 6f, g), indicating a reduction in PKCα activity. Importantly, PKCα activity in the *Acsl4*-deficient hippocampus was significantly restored by PKC activator, Bryostatin 1 (Fig. 6f, g). Together, these results demonstrate an essential role of Acsl4 in the hippocampal DAG-PKC signaling activation.

### Pharmacologic activation of PKC in *Acsl4*-deficient mice

Bryostatin 1, a natural macrolide derived from the bryozoan Bugula neritina, is a potent and central nervous system (CNS)-permeable PKC activator^76^. It has been tested in clinical trials for several types of cancer^77, 78^, Alzheimer’s disease (AD)^79, 80^, and multiple sclerosis (MS) (ClinicalTrials.gov ID: NCT06190912), demonstrating its safety in human. To assess whether Bryostatin 1 could rescue synaptic deficits and cognitive impairments in *Acsl4*^*fl/y*^*::Syn1-cre* mice, we intraperitoneally administered Bryostatin 1 or vehicle in *Acsl4*^*fl/y*^*::Thy1-EGFP::Syn1-cre* or *Acsl4*^*fl/y*^*::Thy1-EGFP* control mice three times per week from their birth to adulthood (Fig. 6h). Imaging analyses following immunohistochemistry revealed a significant increase in spine density in *Acsl4*^*fl/y*^*::Thy1-EGFP::Syn1-cre* mice treated with Bryostatin 1, compared to vehicle- or Bryostatin 1-treated controls (Fig. 6i, j). Additionally, Bryostatin 1 normalized dendritic spine maturation, shifting the proportion from immature thin spines toward mature mushroom spines, a hallmark of functional synapses (Fig. 6k). Behavioral assessments further highlighted the therapeutic efficacy of Bryostatin 1 in rescuing cognitive deficits by neuronal *Acsl4* deficiency. In the MWM test, Bryostatin 1-treated *Acsl4*^*fl/y*^*::Thy1-EGFP::Syn1-cre* mice showed a significant reduction in escape latency during the learning phase, increased time spent in the correct quadrant during the probe trials, and enhanced performance in the reversal phase (Fig. 6l–n). Similarly, in the fear conditioning test, Bryostatin 1-treated *Acsl4*^*fl/y*^*::Thy1-EGFP::Syn1-cre* mice exhibited a marked increase in freezing behavior across training, context recall, and tone recall trials, indicating improved associative memory retention (Fig. 6o–q). Moreover, Bryostatin 1-treated knockout mice displayed enhanced spatial memory performance in the Y-maze test, spending significantly more time exploring the novel arm compared to vehicle-treated knockout mice (Extended Data Fig. 13a, b). Collectively, these findings demonstrate that the PKC activator Bryostatin 1 effectively rescues dendritic spine deficits and cognitive impairments in *Acsl4*-deficient mice, likely via restoration of DAG-PKC signaling. This highlights a potential therapeutic strategy for intellectual disabilities associated with ACSL4 mutations.

## Discussion

Lipid metabolism has long been recognized as a cornerstone of brain structure and function, yet its direct contributions to cognition and neurodevelopmental disorders such as intellectual disability (ID) remain poorly defined^8, 15, 18, 26^. In this study, we establish a causal link between lipid dysregulation and cognitive deficits by focusing on Acsl4, a lipid synthetase mutated in X-linked non-syndromic ID. Through a comprehensive set of molecular, imaging, and behavioral analyses, we demonstrate that neuron-specific deletion of Acsl4 impairs hippocampal development, disrupts synaptic plasticity, weakens engram cell activation, and causes severe learning and memory deficits. Mechanistically, these impairments are attributed to a deficiency in diacylglycerol (DAG)-PKCα signaling, caused by the loss of Acsl4-mediated DAG synthesis. Notably, early intervention using the PKC activator Bryostatin 1 fully rescues both synaptic and cognitive phenotypes, positioning lipid signaling modulation as a viable therapeutic strategy for X-linked ID. More broadly, these results reshape our understanding of how lipid metabolism regulates synaptic plasticity and offer a new framework for developing lipid-targeted interventions to reverse developmental brain dysfunction.

Brain development is a highly orchestrated process governed by critical periods, during which neuronal circuits undergo extensive remodeling, synaptic connections are refined, and molecular signaling pathways shape long-term cognitive function^54–56^. These developmental windows are particularly sensitive to genetic and environmental perturbations, with disruptions often leading to neurodevelopmental and neuropsychiatric disorders^57, 81^. Lipid metabolism plays an essential yet underappreciated role in the critical periods, as neuronal membrane expansion, synaptic formation, and intracellular signaling all depend on dynamic lipid modifications^8, 18, 19, 82^. The molecular and neurobiological mechanisms underlying these processes, however, are still poorly understood. Our findings reveal that Acsl4 expression surges during the early postnatal period, peaking around P14 in the hippocampus---a critical developmental phase marked by synaptogenesis, lipid remodeling, and neural circuit maturation. Functional experiments support this temporal pattern: hippocampal deletion of Acsl4 in early postnatal life (P4) induces pronounced cognitive deficits, whereas its deletion in young adulthood has no significant effect. This striking contrast underscores the idea that Acsl4 is indispensable for establishing cognitive function but is not required for its maintenance.

Like many lipid-metabolizing enzymes, Acsl4 undergoes alternative splicing, generating two isoforms: Acsl4_U, the ubiquitous variant, and Acsl4_B, which is specifically expressed in the brain in both mouse and human^41–43^. Despite prior reports identifying Acsl4_B, its functional significance and localization within the CNS remained unexplored. In this study, we utilized vSLENDR-mediated endogenous tagging, allowing for the first direct visualization of Acsl4_B *in vivo*, revealing its distinct enrichment in dendritic spines. In addition, the “delete-then-rescue” approach employed here by combining both neuron-specific knockout mice and AAV-mediated brain re-expression of specific variant provides a powerful paradigm for dissecting isoform-specific functions, particularly for lipid-metabolizing enzymes, which are often studied in bulk without isoform-level resolution. This methodology not only establishes Acsl4_B as the functionally dominant isoform in the brain but also introduces a framework for investigating other lipid enzymes with multiple isoforms, expanding the potential for targeted gene therapies. Understanding how specific Acsl4 isoforms contribute to synaptic regulation and neurodevelopment may open avenues for isoform-selective interventions in ID and related disorders. Meanwhile, several key questions remain unanswered. First, while our current understanding of Acsl4 function is based on studies of Acsl4_U, the extent to which this knowledge can be extrapolated to Acsl4_B remains unclear. Since the brain-specific isoform exhibits a similar enzymatic activity^83^, its subcellular localization likely underlies its unique neuronal and synaptic functions. Future studies should investigate whether their substrate preferences, regulatory mechanisms, and downstream signaling pathways diverge. Second, the precise mechanisms by which Acsl4_B regulate DAG-PKC signaling at synapses warrant further exploration, particularly whether it exerts non-catalytic scaffolding functions in addition to its enzymatic role. Finally, given that Acsl4_B’s N-terminal domain exhibits transmembrane characteristics predicted by DeepLoc 2.1^84^, future studies should also determine how its membrane interactions influence its neuronal distribution and function. Answering these questions will be essential for fully understanding the molecular basis of Acsl4-related ID and for designing isoform-specific therapeutic strategies.

At the molecular level, our lipidomics and *in vivo* imaging pinpoint DAG-PKCα signaling as a critical downstream effector of Acsl4. We found that Acsl4 deficiency leads to a selective reduction in DAG 32:0, resulting in diminished PKCα activity within hippocampal neurons. Given the established role of PKCα in synaptic plasticity, spine maturation, and memory formation, these findings provide a mechanistic framework linking lipid biosynthesis to neural circuit function. Notably, restoring PKCα activity with Bryostatin 1 not only reverses synaptic and structural abnormalities, but also fully rescues cognitive deficits in Acsl4-deficient mice. This highlights the therapeutic potential of targeting Acsl4 downstream pathways, rather than directly modulating Acsl4 itself—an especially important consideration given Acsl4’s complex biology and roles in other disease contexts.

Among these roles, Acsl4 has been extensively studied as a key regulator of ferroptosis, an iron-dependent form of programmed cell death characterized by lipid peroxidation^85–87^. Ferroptosis has been implicated in a wide range of pathological conditions, including neurodegenerative diseases, cancer, and metabolic disorders such as obesity^88–91^. Interestingly, emerging studies suggest that ferroptosis may also play a role in certain aspects of developmental biology, such as muscle differentiation and avian limb growth^92^, raising the possibility that lipid peroxidation pathways could be involved in neurodevelopment. Given that ferroptosis is closely linked to Acsl4 function, an important question is whether this mechanism contributes to the synaptic and cognitive deficits observed in Acsl4-deficient mice.

Several lines of evidence from our study argue against a significant role for ferroptosis in Acsl4-mediated neurodevelopmental deficits. First, our transcriptomic and lipidomic analyses, together with the data in Extended Data Fig. 14a-d, do not reveal ferroptosis-associated signatures or oxidative stress markers. Second, all prior studies linking Acsl4 to ferroptosis have focused on the ubiquitous isoform Acsl4_U, whereas our results identify the brain-specific Acsl4_B as the dominant isoform for neurodevelopment. Whether Acsl4_B itself can even initiate ferroptosis remains unknown, and given its distinct subcellular localization and lipid metabolism functions in dendrites, its role in ferroptosis is likely to be different from that of Acsl4_U. Third, our successful rescue of neuronal and behavioral phenotypes using Bryostatin 1, which acts through PKC and not ferroptosis pathways, further argues against a ferroptosis mechanism. Thus, while Acsl4 may promote ferroptosis in other tissues or disease contexts, this mechanism appears unlikely to explain the neurodevelopmental deficits observed here.

In addition to impairing synaptic function, Acsl4 deletion triggers a broader network of molecular changes, including upregulation of genes involved in lipid metabolism, immune activation, and inflammation. These transcriptomic alterations are accompanied by increases in specific lipid species, which may reflect accumulated Acsl4 substrates or compensatory shifts in metabolic pathways. Some of these lipid molecules, including free PUFAs and lipid mediators, have known bioactive roles in signaling pathways linked to neuronal excitability, microglial function, and neuroinflammation. Notably, several upregulated genes, such as *Cx3cr1* and *Tlr2/3/4/7* are associated with microglial activation and cytokine signaling^93–97^, suggesting an unexpected immune component in Acsl4-deficient brains. Whether these changes contribute causally to cognitive dysfunction, act as secondary responses to synaptic deficits, or represent compensatory adaptations remains an open question. Regardless, these findings raise the possibility that Acsl4’s functions extend beyond neurons, shaping the broader neuroimmune microenvironment through its influence on lipid signaling.

These insights collectively reshape our understanding of Acsl4 and its roles in the developing brain. While our study centers on its enzymatic function in neurons and its regulation of DAG-PKC signaling, the upregulation of immune and metabolic genes highlights the complex and interconnected nature of lipid homeostasis in brain health. The dual impact of Acsl4 loss—on both neuronal signaling and immune pathways—suggests that future therapeutic strategies may need to consider both dimensions. Importantly, our approach using Bryostatin 1 provides a proof-of-concept for targeting downstream effectors of Acsl4, bypassing potential risks associated with direct Acsl4 activation, such as ferroptosis or unwanted effects in peripheral tissues. This strategy may be broadly applicable not only to patients with ACSL4 mutations but also to other neurological disorders involving lipid dysregulation and PKC signaling.

## Methods

### Mice

All experimental procedures complied with protocols approved by the Institutional Animal Care and Use Committee (IACUC) at Boston Children’s Hospital. Mice were group-housed in Optimice cages under AAALAC-recommended temperature and environmental conditions. The following published mouse lines were utilized: *Acsl4*-floxed mice^98^ (*Acsl4*^*fl/y*^ and *Acsl4*^*fl/fl*^) on a *C57BL/6J* background kindly provided by Dr. Andrew Greenberg; *Syn1-cre* mice (JAX #003966), *Thy1-EGFP* mice (JAX #007788), and wildtype *C57BL/6J* mice (JAX #000664). The following crosses were performed to generate experimental animals: *Acsl4*^*fl/y*^*::Syn1-cre* and *Acsl4*^*fl/+*^*::Syn1-cre* mice were derived from *Acsl4*^*fl/fl*^ × *Syn1-cre* crosses; *Acsl4*^*fl/fl*^*::Syn1-cre* mice were derived from *Acsl4*^*fl/+*^::*Syn1-cre × Acsl4*^*fl/y*^ crosses. The genotyping of *Acsl4*-floxed mice was determined by PCR using mouse tail tip gDNA with the following primers: (forward) 5’-CAGTCTTTGGCTGTAAATTGACTATGTGC-3’ and (reverse) 5’-TGTACCAGTTGCTTGGGAGGAGTACA-3’. To exclude germline recombination caused by *Syn1-cre* mice^99^, additional PCR was performed on all progeny from *Syn1-cre* and *Acsl4*-floxed mice crosses using: (forward) 5’-CAGTCTTTGGCTGT AAATTGACTATGTGC-3’ and (reverse) 5’-ACTTGCCAACCAGAAACATGCATAC-3’. All mice had ad libitum access to food and water and were used at the specified ages indicated in the experimental procedures and figure legends. Both male and female mice were included based on experimental requirements, and all comparisons were conducted with sex- and age-matched littermates.

### *In situ* hybridization

*In situ* hybridization (ISH) was carried out using RNAscope technology with the RNAscope Multiplex Fluorescent Reagent Kit v2 (Advanced Cell Diagnostics). The probes employed were Mm-Acsl4-C1, Mm-Slc32a1-C2, and Mm-Slc17a7-C3, Mm-Acsl4-Exons 3/4-C1 RNAscope probes (Advanced Cell Diagnostics, Cat# 443791, 319191-C2, 416631-C3, and 1087631-C1). Brains were collected from male *C57BL/6J* mice at various developmental stages (E18.5, P2, P7, P14, P28, and P56) and adult *Acsl4*^*fl/y*^ and *Acsl4*^*fl/y*^*::Syn1-cre* mice following decapitation. The tissues were fixed in 10% neutral buffered formalin (NBF) at room temperature (RT) for 32 hours. Fixed samples were washed in 1× PBS for 1 hour at RT, followed by dehydration through a standard ethanol gradient and xylene treatment. The samples were then embedded in paraffin using routine methods and sectioned into 5 μm slices using a microtome (Leica RM2125 RTS). Sections were mounted onto Superfros Plus Microscope Slides (Fisher Scientific). The slides were baked in a dry oven at 60°C for 1 hour, then deparaffinized through two 5-minute xylene incubations, followed by two 2-minute incubations in 100% ethanol at RT. After drying at 60°C for 5 minutes, sections were treated with hydrogen peroxide for 10 minutes at RT and rinsed with distilled water. Target retrieval was performed using RNAscope 1× Target Retrieval Reagent at 99°C for 15 minutes, followed by rinsing with distilled water. Slides were then immersed in 100% ethanol for 3 minutes and dried at 60°C for 5 minutes. Protease Plus treatment was applied to the sections for 30 minutes at 40°C in a HybEZ oven (Advanced Cell Diagnostics), followed by rinsing in distilled water. Probes were hybridized to the sections for 2 hours at 40°C, after which the amplification steps were performed sequentially (AMP1 for 30 minutes, AMP2 for 30 minutes, and AMP3 for 15 minutes). Signal detection was achieved by incubating the sections with HRP reagents for 15 minutes at 40°C, followed by treatment with the respective Opal fluorophore reagents (Opal 690 for C1 [1:500 dilution] and Opal 570 for C2 and C3 [1:1000 dilution], Akoya Biosciences, Cat# FP1488001KT, FP1497001KT) for 30 minutes at 40°C. HRP blocker was applied to the sections for 15 minutes at 40°C. Between each incubation step, the sections were washed twice with Wash Buffer for 2 minutes per wash. Finally, the sections were counterstained with DAPI and mounted using ProLong Gold Antifade Mountant (Thermo Scientific, Cat# P36930).

### *In situ* hybridization combined with immunofluorescence

To enable the detection of both mRNA and proteins, RNAscope and immunofluorescence were performed on the same tissue sections. The probes utilized were Mm-Acsl4-C1 and Mm-Acsl4-Exons 3/4-C1. Antibodies used include mouse anti-NeuN (1:100; Millipore, Cat# MAB377), mouse anti-glial fibrillary acidic protein (GFAP) (1:1000; Sigma-Aldrich, Cat# G3893), rabbit anti-ionized calcium-binding adapter molecule 1 (Iba1) (1:200; FUJIFILM Wako, Cat# 019–19741), rabbit anti-tdTomato (1:1000; Takara Bio, Cat# 632496), Alexa Fluor Plus 647-conjugated donkey anti-mouse IgG (1:200; Thermo Fisher, Cat# A32787), Alexa Fluor 647-conjugated donkey anti-rabbit IgG (1:200; Thermo Fisher, Cat# A31573), and Alexa Fluor 546-conjugated donkey anti-rabbit IgG (1:200; Thermo Fisher, Cat# A10040). Adult male *C57BL/6J* mice and AAV_9_-hSyn-Cre-P2A-tdTomato-injected *Acsl4*^*fl/y*^ mice were transcardially perfused with 1× PBS followed by 10% NBF. The brains were then post-fixed at room temperature (RT) for 32 hours. Tissue pretreatment, including steps up to the hydrogen peroxide treatment, was carried out as described in the *In situ* hybridization section. Target retrieval was performed using RNAscope 1× Co-Detection Target Retrieval Reagent at 99°C for 15 minutes, followed by rinsing with distilled water. Sections were then washed in 1× PBS-T (1× PBS containing 0.1% Tween-20). Primary antibodies (NeuN, GFAP, or Iba1 for *C57BL/6J* mice and tdTomato for AAV_9_-hSyn-Cre-P2A-tdTomato-injected *Acsl4*^*fl/y*^ mice) were applied to the sections and incubated overnight at 4°C. After primary antibody incubation, sections were washed three times in PBS-T for 2 minutes each. Next, the sections were fixed in 10% NBF for 30 minutes at RT, followed by three additional PBS-T washes (2 minutes each). Subsequent steps, including Protease Plus treatment and the fluorescent ISH assay, were performed as described in the *In situ* hybridization section. For *C57BL/6J* mouse sections, the probe Mm-Acsl4-C1 was applied, while for AAV_9_-hSyn-Cre-P2A-tdTomato-injected *Acsl4*^*fl/y*^ mouse sections, the probe Mm-Acsl4-Exons 3/4-C1 was used. Signal detection was achieved with Opal 570 for Mm-Acsl4-C1 and Opal 690 for Mm-Acsl4-Exons 3/4-C1. Following the final ISH HRP blocking step, fluorophore-conjugated secondary antibodies were applied to the sections and incubated for 30 minutes at RT. Sections were then washed three times in PBS-T for 2 minutes each. Finally, the sections were counterstained with DAPI and mounted using ProLong Gold Antifade Mountant (Thermo Scientific, Cat# P36930).

### Immunohistochemistry

Mice were transcardially perfused with 1× PBS, followed by NBF. The brains and livers were post-fixed overnight at 4°C and subsequently dehydrated in 30% sucrose solution prepared in 1× PBS. Tissues were frozen using dry ice and sectioned into 40 μm slices with a Leica microtome (Leica SM2010R). Sections were washed in PBS containing 0.25% Triton X-100 (PBT, pH 7.4) and then incubated in blocking buffer consisting of 3% normal donkey serum (Millipore, Cat# S30-M) in PBT-azide for 2 hours. Primary antibody incubation was performed overnight at RT in blocking buffer. After three PBS washes, sections were treated with secondary antibodies for 1 hour at RT. Following three additional PBS washes, sections were mounted onto gelatin-subbed slides (Southern Biotech, Cat# SLD01-CS) using ProLong Gold Antifade Mountant, with or without DAPI (Thermo Scientific, Cat# P36930, P36931), for imaging and storage. Primary antibodies used for immunohistochemistry include: chicken anti-GFP (1:2000; Aves Labs, Cat# GFP-1010), rabbit anti-tdTomato (1:1000; Takara Bio, Cat# 632496), goat anti-mCherry (1:2000; SICGEN, Cat# AB0040), mouse anti-transferrin receptor 1 (TfR1) (1:100; Santa Cruz, Cat# sc-32272). Secondary antibodies include: Alexa Fluor 488-conjugated donkey anti-chicken IgG (1:200; Jackson ImmunoResearch, Cat# 703-545-155), Alexa Fluor 546-conjugated donkey anti-rabbit IgG (1:200; Thermo Fisher, Cat# A10040), Alexa Fluor 546-conjugated donkey anti-goat IgG (1:200; Thermo Fisher, Cat# A11056), and Alexa Fluor Plus 647-conjugated donkey anti-mouse IgG (1:200; Thermo Fisher, Cat# A32787).

### Nissl staining

Mice were transcardially perfused with 1× PBS followed by 10% neutral buffered formalin (NBF). The brains were post-fixed overnight at 4°C and dehydrated in 30% sucrose prepared in 1× PBS. Brains were then frozen on dry ice and sectioned sagittally at 40 μm using a Leica microtome. Sections were mounted onto Superfrost Plus Microscope Slides (Fisher Scientific) and baked at 55°C for 2 hours. Subsequently, the sections underwent the following treatment steps: 100% ethanol for 3 minutes; 95% ethanol for 3 minutes; 70% ethanol for 3 minutes; distilled water for 30 seconds; 0.5% cresyl violet staining for 20 minutes; distilled water for 30 seconds; 70% ethanol for 2 minutes; 95% ethanol for 3 minutes; 100% ethanol for 1 minute (repeated three times); xylene for 5 minutes (repeated twice). Finally, the sections were mounted using Krystalon Mounting Medium (MilliporeSigma, Cat# 64969). The 0.5% cresyl violet solution was prepared as follows: 0.5 g cresyl violet acetate (Chem-Impex, Cat# 22892) was dissolved in 70 ml distilled water. Subsequently, 20 ml of 100% ethanol and 1.5 ml glacial acetic acid were added. The solution volume was adjusted to 100 ml with distilled water, and the pH was adjusted to 3.5–3.7.

### Image acquisition and analysis

All image acquisition and analysis in the current study were conducted by an individual blinded to genotype and treatment groups.

Dendritic branching analysis was conducted using z-stack images acquired from 60 μm thick mouse brain sections with a confocal microscope (Leica TCS SP8, 40× objective). Parameters such as total dendritic length and the number of branch points were evaluated using ImageJ software (version 1.54f). Additionally, dendrites were traced in 3D with the Simple Neurite Tracer plugin in Fiji. Sholl analysis was performed by counting the intersections of concentric spheres centered on the soma with the traced dendrites at various distances.

For dendritic spine analysis, image stacks were captured from secondary dendritic segments within the stratum radiatum using a confocal microscope (Leica TCS SP8, 60× objective). Maximum intensity projection images of these stacks were used for spine morphology quantification in ImageJ. Dendritic spines were categorized into four subclasses—mushroom, thin, stubby, and filopodium—based on previously established morphological criteria, including spine length, neck, and head characteristics^100, 101^.

Immunohistochemistry images (Figures 3J, 5B, 5J, 6C, 6E, S1D–S1F, S1N–S1Q, S2D–S2F, S7L, and S7M) and Nissl staining images were acquired using a 20× objective on the Olympus VS120 Virtual Slide Microscope (Olympus). Fluorescence intensity of TfR1 immunohistochemistry staining in the hippocampus was quantified using ImageJ.

For EGFP-positive cell quantification in vSLENDR experiments, 1 in 3 hippocampal slices per mouse underwent immunohistochemistry staining with an anti-GFP antibody. Images were captured using a confocal microscope (Leica TCS SP8) with 20×, 40×, or 60× objectives. Total EGFP-positive cells in each mouse were manually counted.

RNAscope images of whole sagittal and coronal brain sections, as well as entire hippocampal regions, were acquired using a 20× objective on the Olympus VS120 Virtual Slide Microscope (Olympus). Higher-resolution images of specific hippocampal regions were captured by z-stack scanning with a confocal microscope (Leica TCS SP8) using a 60× objective. RNAscope image quantification was performed using QuPath software^102^, following the guidelines provided by Advanced Cell Diagnostics (https://acdbio.com/qupath-rna-ish-analysis).

### DNA constructs

To generate AAV-HDR constructs for mEGFP-Acsl4_U and mEGFP-Acsl4_B, we first constructed targeting vectors containing HDR donor template DNAs. Approximately 1 kb homology sequences flanking each side of the target site for Acsl4_U and Acsl4_B were amplified by PCR from *C57BL/6J* mouse genomic DNA using the Phanta Flash Super-Fidelity DNA Polymerase (Vazyme, Cat# P520) following the manufacturer’s instructions. The 5′ and 3′ homology sequences were subsequently cloned into the MluI/BamHI and BsrGI/RsrII restriction enzyme sites, respectively, of a pAAV backbone derived from pAAV-Ef1a-mCherry gifted from Dr. Karl Deisseroth (Addgene plasmid, Cat# 114470). The mEGFP sequence was amplified by PCR from pAAV-HDR-mEGFP-Actin gifted from Dr. Ryohei Yasuda (Addgene plasmid, Cat# 119870) and inserted into the targeting vectors containing the Acsl4_U/Acsl4_B homology sequences using seamless cloning with the ClonExpress Ultra One Step Cloning Kit V2 (Vazyme, Cat# C116). This process yielded the AAV-HDR donor templates for mEGFP-Acsl4_U and mEGFP-Acsl4_B. The sgRNA expression cassette, containing the human U6 promoter and an sgRNA specific for *Acsl4* (target sequence: AGCTTAAGGTTCATAGTGGA), was synthesized (Genscript) and cloned into the MluI restriction enzyme site of the AAV-HDR donor templates.

To generate AAV-hSyn-Acsl4_B-P2A-tdTomato vector, the Acsl4_B-P2A-tdTomato sequence was synthesized (Genscript) and cloned into the BamHI/HindIII restriction enzyme sites of a pAAV backbone derived from pAAV-hSyn-DIO-mCherry gifted from Dr. Bryan Roth (Addgene plasmid, Cat# 50459). Similarly, to generate AAV-hSyn-Acsl4_U-P2A-tdTomato, the Acsl4_U-P2A-tdTomato sequence was amplified by PCR from the AAV-hSyn-Acsl4_B-P2A-tdTomato construct and inserted into the same BamHI/HindIII sites of the pAAV backbone.

For the PKCα sensor constructs, the donor part AAV-hSyn-PKCα-mEGFP was generated by amplifying the PKCα-mEGFP sequence via PCR from CMV-PKCα-mEGFP-N1 gifted from Dr. Ryohei Yasuda (Addgene plasmid, Cat# 112269) and seamlessly cloning it into a pAAV backbone derived from pAAV-hSyn-EGFP gifted from Dr. Bryan Roth (Addgene plasmid, Cat# 50465). The acceptor part, AAV-hSyn-2mCherry-PS, was constructed by amplifying the 2mCherry-PS sequence from CMV-2mCh-PS gifted from Dr. Ryohei Yasuda (Addgene plasmid, Cat# 112268) and cloning it into the BamHI/EcoRI restriction enzyme sites of the same pAAV backbone. All final constructs were sequenced and verified to confirm their accuracy.

### AAV production

The following AAVs were acquired from Addgene: AAV_9_-hSyn-EGFP (Cat# 50465-AAV9, 1 × 10^13^ GC ml^−1^ titer), AAV_9_-hSyn-mCherry (Cat# 114472-AAV9, 1 × 10^13^ GC ml^−1^ titer), AAV_9_-hSyn-Cre-P2A-tdTomato (Cat# 107738-AAV9, 1 × 10^13^ GC ml^−1^ titer), AAV_9_-hSyn-Cre (Cat# 105553-AAV9, 1 × 10^13^ GC ml^−1^ titer).

Additional AAV constructs from Addgene include: The AAV constructs AAV-EF1α-Flpo (Plasmid #55637), AAV-EFS-Cas9 (Plasmid #104588), AAV-cFos-tTA (Plasmid #66794), and AAV-TRE-EGFP (Plasmid #89875). These constructs were serotyped with various AAV coat proteins and packaged in our laboratory, including AAV_9_ (Addgene plasmid, Cat# 112865), AAV_5_ (Addgene plasmid, Cat# 104964), AAV_8_ (Addgene plasmid, Cat# 112864), and AAV_DJ_ (Cell Biolabs, Cat# VPK-420-DJ).

Home-built constructs: The home-built AAV-hSyn-Acsl4_B-P2A-tdTomato construct was serotyped with AAV_9_ coat proteins and packaged by the Viral Core at Boston Children’s Hospital (4 × 10^13^ GC ml^−1^ viral titers). The constructs AAV-HDR-mEGFP-Acsl4_U, AAV-HDR-mEGFP-Acsl4_B, and AAV-hSyn-PKCα-mEGFP were serotyped with AAV_8_ coat protein and packaged in our laboratory. The construct AAV-hSyn-2mCherry-PS was serotyped with AAV_DJ_ coat protein and packaged in our laboratory. Additionally, AAV-hSyn-EGFP, AAV-hSyn-Acsl4_U-P2A-tdTomato and AAV-hSyn-Acsl4_B-P2A-tdTomato were serotyped with AAV_CAP-B10_ (Addgene plasmid, Cat# 175004) coat protein and packaged in our laboratory.

AAV packaging protocol is as below. The AAV packaging process was conducted following established Addgene protocols (https://www.addgene.org/protocols/aav-production-hek293-cells/; https://www.addgene.org/protocols/aav-purification-iodixanol-gradient-ultracentrifugation/). Briefly, HEK293T cells (American Type Culture Collection) were transfected with the helper plasmid (Addgene plasmid, Cat# 112867), a RepCap plasmid, and the plasmid expressing the gene of interest using polyethylenimine. Virus was harvested after 72 hours from both cell lysates and media, and purification was performed using iodixanol gradient ultracentrifugation (STEMCELL Technologies, Cat# 07820). All home-built AAVs achieved titers in the range of 5 × 10^12^ to 5 × 10^13^ GC ml^−1^.

### Stereotaxic and intravenous injection of AAVs

Stereotaxic surgeries were conducted to deliver AAV into the hippocampus of P28 mice, following previously established protocols^103^. In brief, P28 mice were anesthetized with isoflurane and secured on a stereotaxic apparatus (KOPF model 922) equipped with ear bars. After making a small incision to expose the skull, a hole was drilled based on bregma-aligned coordinates. A pulled-glass pipette with a tip diameter of 20–40 μm was used to target the hippocampal regions, and 100 nl of AAV viruses were injected at three sites: CA1 (AP −1.80 mm, DV −1.50 mm, LR ±1.30 mm), DG (AP −1.80 mm, DV −2.10 mm, LR ±1.10 mm), CA3 (AP −1.80 mm, DV −2.20 mm, LR ±2.20 mm). The titers of the AAVs were adjusted to 5 × 10^12^ GC ml^−1^ for injection. The AAV was delivered using a lab-designed air-puff system, with injection speed controlled at 50 nl/min using a micromanipulator (Grass Technologies, Model S48 Stimulator). After each injection, the pipette remained in place for 5 minutes to ensure adequate absorption and distribution of the virus before withdrawal. Prior to surgery, analgesics, including Ethiqa XR (3.25 mg/kg) and Buprenorphine HCl (0.1 mg/kg), were administered subcutaneously.

Stereotaxic surgeries for AAV injections at P4 were performed following a previously established protocol^104^. Briefly, EMLA cream (2.5% lidocaine and 2.5% prilocaine) was applied 15 minutes prior to the procedure. P4 mouse pups were anesthetized via hypothermia and secured on a custom-built holder compatible with the base of a stereotaxic apparatus. A small incision was made to expose the skull, and the lambda landmark was identified. Using lambda-aligned coordinates, a hole was drilled at the appropriate site. A pulled-glass pipette with a tip diameter of 20–40 μm was used to target the hippocampus with the following coordinates: AP +1.20 mm, DV −1.80 mm, and LR ±1.45 mm. Each site was injected with 200 nl of AAV with titers adjusted to 1 × 10^12^ GC/ml. Following each injection, the pipette was left in place for 2 minutes to ensure proper diffusion before being gradually withdrawn. The incision was closed with 8–0 absorbable sutures. Pups were allowed to recover either by being held in a gloved hand or placed on a warm water-circulating heating pad before being returned to their dam.

Intravenous AAV injections in neonatal mice at a dose of 1 × 10^12^ viral genomes per animal were performed following a previously established protocol^105^. Briefly, P1 mouse pups were anesthetized using hypothermia and positioned under a microscope. A syringe containing 50 μl of AAV_CAP-B10_ (titer adjusted to 2 × 10^13^ GC/ml) was used to inject the temporal vein with the needle bevel facing upward. Proper needle insertion was confirmed by observing blood filling the needle bevel through the skin. The plunger was depressed slowly, and blanching of the vein along the side of the face was noted. To prevent backflow of the injectant, the needle was left in place for an additional 10–15 seconds before removal. Gentle pressure was then applied to the injection site with a cotton swab until clotting occurred. After the procedure, pups were allowed to recover by being held in a gloved hand or placed on a warm water-circulating heating pad before being returned to their dam. Maternal behaviors such as carrying, bedding, and pup retrieval were closely monitored post-injection.

Following surgery, animals were closely monitored for one week to ensure proper recovery, including regular assessments of body weight and overall health. Injection site accuracy and efficacy were confirmed using electrophysiological microscopy for electrophysiology studies or immunohistochemistry for behavioral studies. Animals with inaccurate or partially effective injections were excluded from the data analysis.

### Drug used *in vivo*

#### Imidazole ketone erastin

A 1.2 mM stock solution of Imidazole Ketone Erastin (IKE) (Cayman Chemical, Cat# 27088) was prepared by dissolving the compound in DMSO and subsequently diluting it with PBS to a concentration of 400 μM. A volume of 150 nl of the 400 μM IKE solution was injected into the hippocampal DG region using the method described earlier, with an equal volume of vehicle injected into the contralateral hippocampus. Four hours post-surgery, the mice were sacrificed, and immunohistochemistry using the TfR1 antibody was performed to detect ferroptosis signals, as described above.

#### Bryostatin 1

Bryostatin 1 treatment in mice was carried out as detailed in prior studies^106^. Bryostatin 1 (MilliporeSigma, Cat# 203811) was initially dissolved in 100% ethanol, then further diluted with PBS containing 20% ethanol and 1% DMSO to achieve a final concentration of 10 μg/mL. Neonatal mice (P1) received intraperitoneal injections of Bryostatin 1 at a dose of 30 μg/kg or an equivalent volume of vehicle control three times per week (Monday, Wednesday, and Friday). The treatment continued until the mice reached adulthood (P56), after which behavioral tests were conducted.

### Behavioral tests

Mice were acclimated to the testing room for 30–60 minutes prior to each test. Data acquisition and analysis were conducted by an individual blinded to genotype and treatment groups. All experiments were performed on adult mice aged 2–3 months.

#### Morris water maze test

The Morris water maze test was conducted as described previously^107^. A white, opaque circular tub (60 cm deep × 83 cm in diameter) was filled with water maintained at approximately 25°C to a depth of 29 cm. Four distinct visual markers were placed on the inner walls of the tub to define four quadrants. Trials were recorded and analyzed using EthoVision XT video tracking software (v.12.0, Noldus Information Technologies). Acquisition training (Day 1): Each mouse underwent eight trials with a visible platform (10 cm in diameter), positioned 0.5 cm above the water surface and marked with a red reflector. Mice were released near the pool’s edge, with starting locations alternated semi-randomly, ensuring each mouse began twice in each quadrant. Hidden training (Days 2–3): Mice completed 20 trials (12 on Day 2 and 8 on Day 3) with the platform submerged 1 cm below the water surface in a new quadrant. Starting locations alternated so each mouse began five times in each quadrant. Probe test (Day 4): The platform was removed, and each mouse completed four 90-second trials, starting from a different quadrant each time, allowing them to explore freely. Reversal training (Day 5): The platform was relocated to a new quadrant and submerged 1 cm below the water surface. Each mouse completed four trials, starting opposite the new platform’s location. Mice had a maximum of 90 seconds to locate the platform. For all visible, hidden, and reversal trials, mice that failed to find the platform within 90 seconds were guided to it by the experimenter. After reaching the platform, mice were allowed to remain on it for 5 seconds before removal. Between trials, mice were placed in a cage lined with absorbent paper towels for rest.

#### Y-maze test

The Y-maze apparatus is consisted of three arms, each measuring 35 cm in length, 15 cm in height, and 5 cm in width, with distinct visual markers placed at the end of each arm. During the training phase, one arm was blocked using a removable door. Mice were placed in the start arm, facing the center of the maze, and allowed to explore two arms (the start arm and one other) for 10 minutes. Thirty minutes later, the test phase was conducted, during which the door blocking the novel arm was removed. The mice were again placed in the start arm and allowed to explore all three arms for 5 minutes. The time spent in each arm was recorded by Noldus EthoVision XT.

#### Fear conditioning test

The fear conditioning test was performed in a small conditioning chamber enclosed within a sound-attenuating chest (Ugo Basile)^108, 109^. On day 1, mice were placed in the conditioning chamber, a square box with vertical striped wallpaper, for 8 minutes. During this period, a 10 kHz tone was presented for 30 seconds, followed by a foot shock (2 seconds at 0.30 mA). This tone-shock pairing was repeated three times with 2.5-minute intervals. On day 2, mice were returned to the same conditioning chamber for 5 minutes to assess contextual fear memory. Next, mice were placed in a novel chamber with distinct contexts—a circular box with black-and-white spotted wallpaper—for 5 minutes, followed by a 3-minute tone presentation to assess cued fear memory. Freezing responses were recorded during all tests and analyzed using EthoVision XT software.

#### Hot plate test

The metal hot plate was heated to 52°C, and mice were gently placed on its surface. The withdrawal latency was manually recorded until the mouse displayed a nociceptive response, such as hind paw flicking, licking, or jumping.

#### Startle response and prepulse inhibition test

The startle reflex measurement system (Kinder Scientific) was utilized to evaluate the startle response and prepulse inhibition (PPI) as previously reported^108, 110^. During each session, the mouse was placed in a chamber and allowed to acclimate for 5 minutes. Startle responses were recorded over a 250-ms period, beginning with the onset of the stimulus. The chamber maintained a background noise level of 60 dB. Peak startle amplitude within the 250-ms window served as the measure of the startle response. The startle response test was performed on day 1, during which each mouse was exposed to a block of nine noise trials. This block was repeated five times, resulting in a total of 45 trials. The inter-trial interval ranged from 10 to 20 seconds. One trial measured the startle response to background noise at 60 dB, while the remaining eight trials assessed responses to 40-ms sound bursts at intensities of 70, 80, 85, 90, 95, 100, 105, and 115 dB. The nine trial types were presented in a pseudorandom order, ensuring each type appeared once within a block. On day 2, the PPI test was performed. In this test, a 20 ms prepulse sound (70, 74, 78, or 82 dB) was presented 100 ms before the startle stimulus (105 dB). The four distinct prepulse stimuli were presented in a pseudorandom order within a single trial block. This block was repeated five times, resulting in a total of 20 trials. The intertrial interval ranged from 10 to 20 seconds. The percentage of PPI was calculated using the formula: %PPI = 100 – [100 × (Startle response on prepulse trials/Startle response on 105 dB startle trials)].

### Engram cells labeling

To label engram cells in the hippocampus associated with fear memory^111, 112^, mice at P28 were bilaterally microinjected with 200 nl of a viral mixture containing AAV_8_-cFos-tTA and AAV_DJ_-TRE-EGFP into the CA1 region using the method described above. Following the virus injection, the mice were maintained on a Doxycycline (Dox)-supplemented diet (200 mg/kg; Bio-Serv, Cat # S3888) for three weeks. After the three-week Dox diet, the Dox-containing food was replaced with regular chow for approximately 24 hours to allow for activity-dependent labeling. After this Dox-free period, the mice underwent fear conditioning training as described earlier to induce neuronal activity. Following the fear conditioning, the mice were returned to their home cages and given a Dox diet. On the next day, fear memory recall tests were conducted, followed by electrophysiological recordings one hour later.

### Acute brain slice preparation

To prepare brain slices, mice aged 7–8 weeks were anesthetized with isoflurane and perfused intracardially with oxygenated, ice-cold dissection buffer containing the following (in mM): 82.75 NaCl, 2.4 KCl, 6.8 MgCl_2_, 0.5 CaCl_2_, 1.4 NaH_2_PO_4_, 23.8 NaHCO_3_, 23.7 -glucose, and 65 sucrose. Coronal hippocampal slices, 300 μm thick, were prepared using a vibratome (VT1000S; Leica Microsystems, Germany) and equilibrated for 1 hour at 31°C in artificial cerebrospinal fluid (aCSF) containing (in mM): 125 NaCl, 2.5 KCl, 1 MgCl_2_, 2 CaCl_2_, 1.25 NaH_2_PO_4_, 25 NaHCO_3_, and 10 -glucose, with osmolality adjusted to 300–310 mOsm·kg^−1^ using sucrose. Subsequently, the slices were transferred to RT for electrophysiological recordings and two-photon microscopy imaging. Throughout the procedure, all buffers were continuously bubbled with a 95% O_2_ / 5% CO_2_ gas mixture.

### Electrophysiological recordings

#### Whole-cell patch-camp recordings

For brain slice whole-cell patch-clamp recordings, individual slices were placed in a recording chamber and continuously perfused with oxygenated aCSF at room temperature. Slices were visualized using an upright epifluorescence microscope equipped with differential interference contrast optics and an infrared CCD camera. All recordings were conducted in the hippocampal CA1 region using a MultiClamp 700B amplifier (Molecular Devices). AAV-infected CA1 neurons were identified by detecting red (mCherry/tdTomato), green (GFP), or both types of fluorescence under epifluorescence illumination. Signals were low-pass filtered at 2 kHz and sampled at 10 kHz using a Digidata 1550B digitizer (Molecular Devices). Patch pipettes with a resistance of 3–5 MΩ were pulled from borosilicate glass capillaries (World Precision Instruments, Cat# 1B150F3) using a microelectrode puller (Model P-1000, Sutter Instruments). For mEPSC/mIPSC and EPSC paired-pulse ratio (PPR) recordings, the internal solution contained (in mM): 132.5 Cs-gluconate, 17.5 CsCl, 2 MgCl_2_, 0.5 EGTA, 10 HEPES, 4 Mg-ATP, and 5 QX-314 chloride (280–300 mOsm·kg^−1^, pH 7.2 adjusted with CsOH). For mEPSC recordings, CA1 neurons were voltage-clamped at −70 mV in the presence of TTX (0.5 μM) and picrotoxin (100 μM) without stimulation. For mIPSC recordings, neurons were held at 0 mV with the application of NBQX (10 μM), D-APV (50 μM), and TTX (0.5 μM). Both mEPSCs and mIPSCs were analyzed using MiniAnalysis software (Synaptosoft). For PPR recordings, CA1 neurons were voltage-clamped at −70 mV, and EPSCs were evoked by two consecutive pulses with interpulse intervals of 25, 50, 75, 100, or 200 ms using a concentric bipolar stimulating electrode positioned at the CA1 stratum radiatum. PPR was calculated as the ratio of the second response peak to the first response peak. To measure the excitability of CA1 neurons, recordings were performed in current-clamp mode using an internal solution containing (in mM): 145 K-gluconate, 5 NaCl, 10 HEPES, 2 Mg-ATP, 0.1 Na_3_GTP, 0.2 EGTA, and 1 MgCl_2_ (280–300 mOsm·kg^−1^, pH 7.2 adjusted with KOH). Action potentials were elicited by applying 500 ms stepwise current injections ranging from 0 to 300 pA. The initial access resistance of recorded neurons was 10–25 MΩ and was monitored throughout the experiment. Data were excluded if the access resistance changed by more than 20% during the recording.

#### Multichannel field potential recordings

A 64-channel multisite recording system (MED64, Alpha Med Scientific) was used to perform extracellular field potential recordings. The MED64 probe (Alpha Med Scientific, Cat# MED-P515A) was prepared according to standard protocols as previously described^62, 63^. Slice preparation followed the procedures outlined earlier, with acutely prepared hippocampal slices allowed to recover in oxygenated aCSF at 31°C for 2 hours. Each slice was then placed on the MED64 probe, ensuring the hippocampal CA1 region was fully aligned with the recording array. Slices were continuously perfused with oxygenated, fresh aCSF during the experiments. After a recovery period of 10–15 minutes on the probe, a microelectrode positioned over the CA1 stratum radiatum was selected for stimulation. Monopolar, biphasic constant-current pulses (0.2 ms duration) were delivered at 0.017 Hz via the selected electrode using the data acquisition software Mobius (Alpha Med Scientific). Field excitatory postsynaptic potentials (fEPSPs) recorded by the remaining microelectrodes were amplified through a 64-channel amplifier, displayed on a monitor, and saved to a microcomputer for offline analysis. For long-term potentiation (LTP) induction, baseline synaptic responses were stabilized for at least 20 minutes before applying a theta burst stimulation (TBS) protocol: 5 bursts at 5 Hz, repeated 5 times at 10-second intervals, with 4 pulses at 100 Hz per burst. The stimulation intensity was set to elicit 40%–60% of the maximum response. For long-term depression (LTD) induction, baseline responses were recorded for at least 15 minutes, followed by delivery of a low-frequency stimulation (LFS) protocol consisting of 900 pulses at 1 Hz. Multichannel electrophysiological data were analyzed offline using MED64 Mobius software. Channels with an fEPSP amplitude greater than −20 μV were defined as activated channels. The slope of fEPSPs for each activated channel was measured, normalized, and expressed as a percentage change from baseline for LTP and LTD quantification. Activated channels in which the average fEPSP slope during the last 5 minutes of recording increased by at least 20% from baseline were classified as LTP channels, while those in which the average fEPSP slope decreased by at least 20% from baseline were classified as LTD channels. The number of activated channels showing LTP or LTD was counted, and the induction ratio was calculated using the formula: Induction ratio = (Number of LTP or LTD channels/Number of all activated channels) × 100%.

### Tissue dissection for omic studies

As described above, *Acsl4*^*fl/y*^ mice at P4 were injected with AAV_9_-hSyn-Cre-P2A-tdTomato in one hippocampus and AAV_9_-hSyn-EGFP in the contralateral hippocampus as a control. To account for potential hemisphere-specific effects, we alternated the injection sites: half of the mice received AAV_9_-hSyn-Cre-P2A-tdTomato in the left hippocampus and AAV_9_-hSyn-EGFP in the right, while the other half received the reversed arrangement. In adulthood, the mice were decapitated under isoflurane anesthesia, and the virus-infected hippocampal tissue was rapidly dissected on ice under a fluorescent stereomicroscope (Zeiss Stereo Discovery V20).

### RNA sequencing (RNA-Seq) and analysis

Total RNA was extracted from collected hippocampal tissues using the RNeasy Plus Mini Kit (Qiagen) following the manufacturer’s instructions. RNA-Seq libraries were generated using the Illumina TruSeq Stranded mRNA Library Prep Kit (Illumina, San Diego, CA). In brief, 500 ng of total RNA was used to enrich poly(A) mRNA through oligo(dT) magnetic bead selection. The enriched mRNA was fragmented at 94°C and reverse transcribed into cDNA using random hexamers and SuperScript II Reverse Transcriptase. To maintain strand specificity, second-strand synthesis incorporated dUTP in place of dTTP. The cDNA was then end-repaired, 3’-adenylated, and ligated to Illumina sequencing adapters. Strand specificity was maintained during library preparation by amplifying only the cDNA strand containing incorporated dUTP through PCR. Final libraries were quantified, assessed for quality using a Tapestation 4200 (Agilent Technologies), and sequenced on an Illumina NovaSeq6000 S4 platform with the NovaSeq 6000 S4 Reagent Kit v1.5 (300 cycles). Raw sequencing counts were normalized, and differential expression analysis was conducted using the DESeq2 package^113^ in R. Heatmaps were generated using the “pheatmap” R function. Gene Ontology (GO) analysis was performed using the clusterProfiler package^114^ in R.

### Untargeted lipid profiling and analysis

C18-negative ion mode lipid profiling: Fatty acids were measured using reversed-phase C18 chromatography coupled with negative ion mode mass spectrometry (MS) on an LC-MS system comprising a Shimadzu Nexera X2 U-HPLC (Shimadzu Corp.) connected to a Q-Exactive Orbitrap mass spectrometer (Thermo Fisher Scientific). Hippocampus was homogenized 1:9 (weight:vol) with water using the QIAGEN TissueLyser II with 2 × 3 mm tungsten beads for 4 minutes at 20 Hz. 30uL of homogenate was precipitated with 90uL methanol containing 50 ng/mL internal standards (15-methyl PGE1, 15-methyl PGA2, and 15-methyl PGE2, Cayman Chemical Co.). After centrifugation at 15,000×g for 10 minutes at 4°C, 2 μL of the supernatant was injected onto a 150 × 2.1 mm, 1.8 μm ACQUITY HSS T3 C18 column (Waters). The column was equilibrated isocratically with 80% mobile phase A (0.01% formic acid in water) for 3 minutes, followed by a linear gradient to 100% mobile phase B (0.01% acetic acid in acetonitrile) over 12 minutes. MS analysis was conducted using electrospray ionization in negative ion mode with full scan detection across 70–850 m/z at a resolution of 70,000 and a data acquisition rate of 3 Hz. Additional MS parameters included: sheath gas at 45, in-source CID at 5 eV, sweep gas at 10, spray voltage at −3.5 kV, capillary temperature at 320°C, S-lens RF at 60, probe heater temperature at 300°C, 1 microscan, automatic gain control target of 1e6, and a maximum ion time of 250 ms. Raw data were analyzed using TraceFinder software (Thermo Fisher Scientific) for targeted peak integration and manual review of identified metabolites, as well as Progenesis QI (Nonlinear Dynamics) for peak detection and integration of both known and unknown metabolites.

C8-positive ion mode lipid profiling: Reversed-phase C8 chromatography coupled with positive ion mode MS detection was employed to measure lipids. Polar and non-polar lipids were analyzed using an LC-MS system comprising a Shimadzu Nexera X2 U-HPLC (Shimadzu Corp.) and an Exactive Plus Orbitrap mass spectrometer (Thermo Fisher Scientific). 10uL of Hippocampal tissue-homogenate was extracted using 190 μL of isopropanol containing 1,2-didodecanoyl-sn-glycero-3-phosphocholine (Avanti Polar Lipids) as an internal standard. Following centrifugation (10 minutes at 10,000 × g), 2uL of the supernatant was injected onto a 100 × 2.1 mm, 1.7 μm ACQUITY BEH C8 column (Waters). The column was initially eluted isocratically with 80% mobile phase A (95:5:0.1 vol/vol/vol 10 mM ammonium acetate/methanol/formic acid) for 1 minute, followed by a linear gradient to 80% mobile phase B (99.9:0.1 vol/vol methanol/formic acid) over 2 minutes and further to 100% mobile phase B over 7 minutes. This was followed by a 3-minute hold at 100% mobile phase B. Mass spectrometry analyses were conducted using electrospray ionization in positive ion mode, with full-scan analysis over 200–1100 m/z at a resolution of 70,000 and a 3 Hz data acquisition rate. Additional MS parameters included: sheath gas 50, in-source CID 5 eV, sweep gas 5, spray voltage 3 kV, capillary temperature 300°C, S-lens RF 60, heater temperature 300°C, microscans 1, automatic gain control target 1e6, and maximum ion time 100 ms. Raw data were processed using TraceFinder software (Thermo Fisher Scientific) for targeted peak integration, with manual review of a subset of identified lipids. Progenesis QI (Nonlinear Dynamics) was employed for peak detection and integration of both identified and unknown lipids. Lipid identities were confirmed by comparison to reference plasma extracts and denoted by the total number of carbons and double bonds in the lipid acyl chains.

For the differential analysis, all data of identified lipids were normalized with internal standards. To identify differentially regulated lipids between the *Acsl4*-deleted and control hippocampi, we used limma R package^115^ by applying a paired t-test with a significance threshold of *P* < 0.05, along with a log_2_(fold change) cutoff of > 0.25 or < −0.25. Volcano plots and heatmaps were generated in R to visualize the lipid profiles.

### HEK293T cell culture and transfection

HEK293T cells were seeded on circular cover glasses (Fisher Scientific, Cat# 12-541-002) and maintained in Dulbecco’s Modified Eagle Medium (DMEM; Thermo Scientific, Cat# 11995073) supplemented with 10% fetal bovine serum (FBS; Corning, Cat# 35–011-CV) at 37°C in a 5% CO_2_ atmosphere. Transfection with PKCα sensor plasmids was performed using Lipofectamine 3000 (Thermo Scientific), with a plasmid ratio of 1:2 for the donor to acceptor constructs.

### Two-photon fluorescence lifetime imaging (2P-FLIM) and analysis

2P-FLIM was conducted using a customized two-photon microscopy system (FEMTO3D Atlas, Femtonics) to measure PKCα activity in HEK293T cells and brain slices. Briefly, mEGFP was excited using a Mai Tai HP laser (MSK Spectra-Physics) at a wavelength of 920 nm. Fluorescence was collected through a 60× objective lens (1.1 N.A., Olympus LUMFL N), split with a shortpass dichroic mirror (565 nm, Chroma, Cat# T565spxr), and detected by a FLIM hybrid detector (HPM-100, Becker & Hickl) positioned after a wavelength filter (Chroma, Cat# ET520/60m). Fluorescence lifetime images were acquired using a time-correlated single-photon counting module (SPC-QC-104, Becker & Hickl), controlled by MES software (Femtonics) in MATLAB and SPCM data acquisition software (Becker & Hickl). FLIM imaging was performed using raster scanning under AO 2P mode at 512 × 512 pixels resolution with the scan speed 1.

For HEK293T cells, imaging was carried out at room temperature 24–48 hours post-transfection. Glass coverslips containing HEK293T cells were placed in a recording chamber on the microscope stage. The chamber was continuously perfused with aCSF bubbled with 95% O_2_ / 5% CO_2_. Baseline images were recorded for at least 10 minutes before stimulation. Cells were then stimulated with 1 μM Phorbol 12,13-dibutyrate (PDBu) (MedChem, Cat# HY-18985) followed 2.5 minutes later by 1 μM ionomycin (Sigma-Aldrich, Cat# I0634). FLIM images were captured every minute for a total of 25 minutes throughout the procedure.

Coronal hippocampal slices were prepared from mice injected with AAVs carrying PKCα sensors (a mixture ratio of 1:2 for the donor to acceptor AAVs), as described earlier. The hippocampal slices were transferred to a recording chamber continuously perfused with aCSF bubbled with 95% O_2_ / 5% CO_2_. Baseline FLIM images of the CA1 region were captured for at least 10 minutes, with one image per minute. When indicated, slices were stimulated with 1 μM PDBu and 2.5 minutes later with 1 μM ionomycin or 1 μM Bryostatin 1. FLIM images were recorded every minute for a total of 25 minutes during the experiment.

All FLIM images were analyzed using SPCImage NG data analysis software (SPCImage, Becker & Hickl), following the manufacturer’s instructions (opm-SPCImage-brochure-v07, Becker & Hickl). Briefly, fluorescence decay curves for each pixel in the FLIM images were obtained using the “decay matrix” function. Then regions of interest (ROIs) were defined using the “mask” function, and the average fluorescence lifetimes within the ROIs were calculated.

### Statistical analysis

Statistical analyses were conducted using GraphPad Prism 9. Parametric tests, such as the two-tailed unpaired t-test, paired t-test, and one-way ANOVA, were applied when data distributions met normality criteria based on the Kolmogorov–Smirnov, Shapiro-Wilk, or D’Agostino & Pearson tests. If normality assumptions were not met, nonparametric tests, such as the Mann-Whitney test, were used. Post hoc comparisons following one-way ANOVA were performed using Dunnett’s or Tukey’s multiple comparisons test, as recommended by Prism. For analyses involving multiple groups and variables, two-way ANOVA was applied without formal normality testing, followed by Sidak’s or Tukey’s multiple comparisons test based on Prism recommendations. Statistical significance thresholds were defined as *P < 0.05, **P < 0.01, ***P < 0.001, and ****P < 0.0001. Error bars represent the mean ± SEM. Comprehensive data and statistical details, including exact P values, F values, t values, degrees of freedom, and sample sizes (n), are provided in Source Data.

## Supplementary Material

Supplementary Files

This is a list of supplementary files associated with this preprint. Click to download.

- SupplementaryTable2.Untargetedlipidomicsrawdata.xlsx
- SupplementaryTable1.RNAseqrawcount.xlsx
- ExtendedDataLi.docx

## Data Availability

All data generated or analyzed during this study are included in the paper and supplemental information. The raw RNA sequencing and lipidomics data in this study will be deposited in Gene Expression Omnibus and MetaboLights. Source Data are provided with this paper.
